# Urinary Metabolite Profiling to Non-Invasively Monitor the Omega-3 Index: An Exploratory Secondary Analysis of a Randomized Clinical Trial in Young Adults

**DOI:** 10.3390/metabo13101071

**Published:** 2023-10-12

**Authors:** Brittany C. MacIntyre, Meera Shanmuganathan, Shannon L. Klingel, Zachary Kroezen, Erick Helmeczi, Na-Yung Seoh, Vanessa Martinez, Adrian Chabowski, Zeny Feng, Philip Britz-McKibbin, David M. Mutch

**Affiliations:** 1 Department of Human Health and Nutritional Sciences, University of Guelph, Guelph, ON N1G 2W1, Canada; bmacin02@uoguelph.ca (B.C.M.); shannonklingel@gmail.com (S.L.K.); 2 Department of Chemistry and Chemical Biology, McMaster University, Hamilton, ON L8S 3W3, Canada; shanmm2@mcmaster.ca (M.S.); kroezezp@mcmaster.ca (Z.K.); helmecwe@mcmaster.ca (E.H.); seohn@mcmaster.ca (N.-Y.S.); martiv4@mcmaster.ca (V.M.); 3 Department of Physiology, Medical University of Bialystok, 15-222 Bialystok, Poland; adrian.chabowski@umb.edu.pl; 4 Department of Mathematics & Statistics, University of Guelph, Guelph, ON N1G 2W1, Canada; zfeng@uoguelph.ca

**Keywords:** precision nutrition, metabolomics, omega-3 long-chain polyunsaturated fatty acids (n3-LCPUFA), omega-3 index, dietary biomarkers, urinary metabolites

## Abstract

The Omega-3 Index (O3I) reflects eicosapentaenoic acid (EPA) and docosahexaenoic acid (DHA) content in erythrocytes. While the O3I is associated with numerous health outcomes, its widespread use is limited. We investigated whether urinary metabolites could be used to non-invasively monitor the O3I in an exploratory analysis of a previous placebo-controlled, parallel arm randomized clinical trial in males and females (*n* = 88) who consumed either ~3 g/d olive oil (OO; control), EPA, or DHA for 12 weeks. Fasted blood and first-void urine samples were collected at baseline and following supplementation, and they were analyzed via gas chromatography and multisegment injection–capillary electrophoresis–mass spectrometry (MSI-CE-MS), respectively. We tentatively identified *S*-carboxypropylcysteamine (CPCA) as a novel urinary biomarker reflecting O3I status, which increased following both EPA and DHA (*p* < 0.001), but not OO supplementation, and was positively correlated to the O3I (R = 0.30, *p* < 0.001). Additionally, an unknown dianion increased following DHA supplementation, but not EPA or OO. In ROC curve analyses, CPCA outperformed all other urinary metabolites in distinguishing both between OO and EPA or DHA supplementation groups (AUC > 80.0%), whereas the unknown dianion performed best in discriminating OO from DHA alone (AUC = 93.6%). Candidate urinary biomarkers of the O3I were identified that lay the foundation for a non-invasive assessment of omega-3 status.

## 1. Introduction

The omega-3 long-chain polyunsaturated fatty acids (n3-LCPUFA) eicosapentaenoic acid (EPA) and docosahexaenoic acid (DHA) have recognized anti-inflammatory and triglyceride-lowering properties [1,2] and are associated with reductions in coronary heart disease mortality [3], neuropsychiatric disorders [4], and all-cause mortality [5]. EPA and DHA are primarily obtained through the consumption of fatty fish and other marine foods, but small amounts are also produced endogenously [6,7]. Due to the numerous health benefits associated with n3-LCPUFA, monitoring their levels in the population is important to help prevent and/or mitigate health risks. 

The two most common methods to determine a person’s n3-LCPUFA status are through dietary assessment or through directly measuring their levels in the blood; however, both methods have recognized limitations. Self-reported assessments of food intake, such as food frequency questionnaires and diet records, are subject to reporting biases [8,9] and are unable to account for individual differences in n3-LCPUFA digestion, absorption, and metabolism that ultimately affect their levels in the body. The current gold standard to determine a person’s n3-LCPUFA status is to directly measure EPA and DHA levels in erythrocytes using gas chromatography–flame ionization detection (GC-FID) or mass spectrometry [10]. The sum of EPA and DHA in erythrocytes is commonly known as the Omega-3 Index (O3I). In contrast to fatty acids measured in serum, plasma, or whole blood, the O3I is less sensitive to acute dietary changes and was reported to reflect n3-LCPUFA membrane content in other tissues such as heart, liver, muscle, and kidney [11,12]. Consequently, the O3I has been positioned as a modifiable and diet-sensitive biomarker that can be used to estimate cardiovascular disease (CVD) mortality and other health risks, where an O3I value of <4% corresponds to high risk and >8% corresponds to low risk [13]. However, a major limitation with measuring the O3I is the need for a blood sample, which presents a challenge in many people due to needle phobia [14,15], as well as the lack of a globally accepted standardized methodology for calculating the O3I [16].

To overcome the challenges associated with dietary assessments, blood sampling, and complicated sample workup procedures, researchers have begun to explore the use of urinary metabolites to objectively and non-invasively monitor an individual’s dietary intake and/or nutrient status [17,18,19]. Several studies have proposed candidate urinary biomarkers related to fish intake, including acetylcarnitine, methylbutyrylcarnitine, propionylcarnitine, and 3-methylhistidine [20]; however, these urinary metabolites remain to be independently validated and may not be specific to n3-LCPUFA. The O3I was recently reported to be inversely associated with the urinary albumin–creatinine ratio [21] and 8-hydroxy-2′-deoxyguanosine (8-OHdG; [22]), while other studies have reported urinary metabolites that change following n3-LCPUFA supplementation, such as trimethylamine-*N*-oxide (TMAO) [23] and 3-carboxy-4-methyl-5-propyl-2-furanpropanoic acid (CMPF) [24]. However, no study to date has investigated if urinary metabolites could serve as a biomarker for the O3I. As such, identifying and validating urinary metabolites that reflect an individual’s O3I could potentially lay the foundation for the development of a non-invasive biomonitoring strategy for precision nutrition. 

The present study is an exploratory secondary analysis of a previously reported randomized control trial in which the differential effects of high-dose EPA and DHA supplementation on the O3I and blood pressure regulation were investigated [25]. The current analysis aimed to identify and evaluate candidate urinary metabolites that can serve as non-invasive biomarkers of the O3I in a cohort of healthy young adults who consumed EPA or DHA supplements for 12 weeks. We hypothesized that a small number of urinary metabolites would be responsive and specific to changes in n3-LCPUFA status such that they could perform reliably as a non-invasive biomarker of the O3I. An untargeted data workflow was used to characterize changes in the urine metabolome via multisegment injection–capillary electrophoresis–mass spectrometry (MSI-CE-MS) and identify novel biomarkers temporally associated with EPA and/or DHA intake.

## 2. Materials and Methods

### 2.1. Participants and Study Design

Ninety healthy men and women aged 18–30 years were recruited from the University of Guelph (Guelph, Ontario, Canada) between January 2017 and June 2017. Exclusion criteria included smoking, history of cardiovascular disease, chronic use of pharmacological medications (except for oral contraceptives), >2 servings of fish or shellfish (or other EPA/DHA fortified foods) per week, and fish oil supplementation in the previous 3 months. All participants provided written informed consent, and the study was approved by the Research Ethics Board at the University of Guelph. The trial was registered at clinicaltrials.gov (NCT03378232).

Eighty-eight participants completed the 12-week double-blind, parallel arm, randomized trial in which the effects of ~3 g/day olive oil (OO; control), EPA, or DHA supplementation were compared. Participants were randomized into one of 3 groups, (1) OO supplement, (2) EPA supplement, and (3) DHA supplement, with block randomization stratified by sex. Participants were instructed to maintain previous dietary and exercise habits for the duration of the trial and one day prior to blood and urine collection [25]. Following an overnight fast, blood and first-void urine samples were collected before (baseline) and after the 12-week supplementation period and stored at −80 °C until analysis. Participant characteristics, including age, height, and weight, were measured at baseline. 

### 2.2. Supplements

Olive oil and purified EPA and DHA (KD-PUR DHA700TG) oil supplements were obtained from KD Pharma (Bexbach, Germany) and encapsulated using InnovaGel in identical softgel capsules. Fatty acid (FA) purity (mean ± SEM) was determined via gas chromatography and previously reported to be 75.7 ± 0.1% for oleic acid (18:1n-9) in the olive oil supplement, 74.7 ± 0.09% EPA and 0.55 ± 0.01% DHA in the EPA oil supplement, and 72.3 ± 1.3% DHA and 1.05 ± 0.11% EPA in the DHA oil supplement [26]. Quantitative fatty acid purity levels were also reported previously [25], with the OO capsules containing 818 mg oleic acid and no detectable EPA or DHA, EPA capsules containing 813 mg EPA and 7 mg DHA, and DHA capsules containing 814 mg DHA and 7.5 mg EPA. Supplementation of 4 capsules/d at these amounts corresponded to approximately 3 g/d of oleic acid, EPA, and DHA. Participants were instructed to take 2 capsules twice daily with food for 12 weeks.

### 2.3. Erythrocyte Fatty Acid Content

Blood samples were collected from the antecubital vein into EDTA-treated vacutainers to isolate erythrocytes. Samples were separated via centrifugation at 700× *g* at 4 °C for 15 min. Erythrocyte samples were shipped to the Medical University of Białystok, Poland, on dry ice for fatty acid composition analysis. Lipids were extracted according to the Folch method [27]. Briefly, lipids from isolated erythrocytes were extracted in chloroform:methanol (2:1 vol:vol) containing butylated hydroxy-toluene (0.01%) as an antioxidant and heptadecanoic acid (17:0) as an internal standard, as previously described [28]. After lipid extraction and transmethylation with BF_3_/methanol, the lipid phase containing fatty acid methyl esters (FAMEs) was dissolved in hexane and analyzed using a Hewlett-Packard 5890 Series II gas chromatograph with a Varian CP-SIL capillary column (100 m, internal diameter 0.25 mm) and flame-ionization detector. Individual fatty acids were detected in accordance with the retention times of standards. FAME and fatty acid standards were purchased from Larodan. A total of 15 FAs were measured: myristic acid (14:0), palmitic acid (16:0), palmitoleic acid (16:1n-7), stearic acid (18:0), oleic acid (C18:1n-9), linoleic acid (18:2n-6), arachidic acid (20:0), α-linolenic acid (ALA; 18:3n-3), behenic acid (22:0), di-homo-γ-linolenic acid (20:3n-6), arachidonic acid (20:4n-6), lignoceric acid (24:0), EPA (20:5n-3), nervonic acid (24:1n-9), and DHA (22:6n-3). FAs were standardized to haemoglobin (Hb) and reported as relative (% FA composition) values or quantitative (ng FA/mg Hb) values. The O3I was calculated through summing the relative % of EPA and DHA.

### 2.4. Untargeted Urine Metabolite Analysis

#### 2.4.1. Chemicals

All chemical standards and calibrants were purchased from Sigma-Aldrich (St. Louis, MO, USA), including analytical grade ammonium acetate, ammonium bicarbonate, ammonium hydroxide, formic acid, organic acids, sodium hydroxide, and recovery/internal standards, including 4-aminobutyric acid-2,2,3,3,4,4-d6 (GABA-d6), choline-d9, 4-fluorophenylalanine (F-Phe), 3-chlorotyrosine (Cl-Tyr), 4-fluorotyrosine (F-Tyr) and naphthalene-2 sulfonic acid (NMS). All LC-MS grade solvents, including acetonitrile, isopropanol, methanol, and water, were obtained from Caledon Laboratories Ltd. (Georgetown, ON, Canada). Calibrant solutions for all analytes were prepared through the serial dilution of stock solutions (50 mM) in LC-MS grade water and stored in a refrigerator (4 °C).

#### 2.4.2. Preparation of Urine Samples and Quality Controls

Briefly, all urine samples were slowly thawed on ice and centrifuged at 10,000× *g* for 5 min. Subsequently, 20 µL of urine was aliquoted into a centrifuge tube and diluted five-fold with 60 µL deionized water, and a 20 µL mixture of internal standard (200 µM Cl-Tyr and NMS) and recovery standards (200 µM GABA-d6, choline-d9, F-Phe, F-Tyr), for a total volume of 100 µL. Centrifuge tubes were vortexed for 5 s; then, 20 µL was aliquoted into a polypropylene vial, and each sample was analyzed in duplicate. Quality controls (QCs) were made to represent the average of all combined samples, to be used to assess instrument drift from run to run. Pooled QCs were prepared through aliquoting 2 µL from each individual sample into a centrifuge tube to establish a representative average sample. Then, a 20 μL aliquot was diluted five-fold with 40 µL deionized water, and 40 µL internal and recovery standard mixture. This QC sample was analyzed in every analytical run via MSI-CE-MS in randomized sample injection positions during data acquisition for the evaluation of technical precision for each urinary metabolite that satisfied our selection criteria (CV < 30% with detection frequency >60%) using a high-throughput MSI-CE-MS metabolomics platform and standardized data workflow for biomarker discovery [29]. Three subgroups of QC samples were also prepared through aliquoting separately from the control, EPA, and DHA supplement arms, which were used for nontargeted metabolite profiling in conjunction with a blank sample. All diluted urine samples were stored at −80 °C prior to analysis.

#### 2.4.3. Urine Metabolome Analysis

An Agilent 6230B time-of-flight (TOF) mass spectrometer with an electrospray ionization (ESI) source equipped to an Agilent G7100A capillary electrophoresis (CE) unit was used for all experiments (Agilent Technologies Inc., Mississauga, ON, Canada). An Agilent 1260 Infinity isocratic pump and a 1260 Infinity degasser were applied to deliver sheath liquid. A sheath liquid composition of 0.1% vol formic acid in (60:40 methanol:water) and (70:30 methanol:water) at a flow rate of 10 μL/min were used for positive and negative ion mode, respectively. For real-time mass correction, purine (20 µL) and hexakis(2,2,3,3-tetrafluoropropoxy)phosphazine (HP-921, 20 µL) reference ions were spiked into the sheath liquid (400 µL) at 0.02% vol to provide constant mass signals. The nebulizer spray was set off during the serial sample injection before being switched on at 4 psi (27.6 kPa) following voltage application. The source temperature was set to 300 °C, and drying gas was delivered at 4 L/min. The instrument was operated under a 2 GHz extended dynamic range for positive and negative modes of detection. The Vcap, fragmentor, skimmer, and octupole RF voltage were set to 3500 V, 120 V, 65 V, and 750 V, respectively. Separations were performed on bare fused-silica capillaries with a 50 μm internal diameter, a 360 μm outer diameter, and a total length of 135 cm (Polymicro Technologies Inc., Phoenix, AZ, USA). A capillary window maker (MicroSolv, Leland, NC, USA) was used to remove 7 mm of the polyimide coating on both ends of the capillary. All diluted urine and pooled QC samples were analyzed via MSI-CE-MS under two different configurations with full data acquisition. Sub-group analysis was also performed via MSI-CE-MS on pooled urine samples from different sub-groups of participants. An acidic background electrolyte (BGE, 1.0 M formic acid with 15% vol acetonitrile, pH 1.8) was used for resolving cationic metabolites under positive ion mode while an alkaline BGE (50 mM ammonium bicarbonate, pH 8.5, adjusted with ammonium hydroxide) was used for anionic metabolites under negative ion mode, as described elsewhere [30,31]. Diluted urine samples were introduced hydrodynamically at 100 mbar (10 kPa), alternating between 5 s for each sample plug and 75 s for each BGE spacer plug, which was electrokinetically injected at 30 kV. In total, thirteen discrete samples were analyzed within a single analytical run of 45 min. The applied voltage was set to 30 kV at 25 °C for CE separations together with a gradient pressure of 2 mbar/min. The structural elucidation of urinary metabolites associated with the O3I was performed via collision-induced dissociation experiments at an optimum collision energy when using a single-injection format in CE coupled to a 6550 quadrupole–time of flight–mass spectrometer system (Agilent Technologies Inc.) under positive or negative ion modes [32]. The structural elucidation of unknown metabolites associated with the O3I was also supported with accurate mass database searches for putative candidate ions reported in the Human Metabolome Database (HMDB 5.0) [33], as well as predicted MS/MS spectra generated in silico via CFM-ID 4.0 [34].

#### 2.4.4. Comprehensive Analysis of Ionic Urinary Metabolites with Quality Control

A targeted and nontargeted data workflow was applied for characterizing urinary metabolites from spurious signals and background ions when using temporal signal pattern recognition with multiplexed separations in MSI-CE-MS [29,35]. An iterative approach was used to authenticate urinary metabolites primarily as their intact molecular ions (i.e., [M + H]^+^ or [M − H]^−^) while filtering out signal redundancy, such as in-source fragments, isotopic contributions, and adducts. Briefly, a sub-group analysis was first performed when using a dilution trend filter comprising 13 independent serial sample injections via MSI-CE-MS under positive and negative ion mode [36], including a quadruple injection of a pooled urine sample comprised of all individuals in the study at both time points together with a single blank sample. An open-source software for data pre-processing of metabolomic data sets, MZmine (version 2.53) [37], was used for reviewing, annotating, and selecting molecular features based on their characteristic accurate mass (*m*/*z*) and relative migration time (RMT) detected under positive (p) or negative (n) ion modes. Also, an in-house urinary metabolite library was used for targeted analysis, where each urinary metabolite was processed using vendor specific software (Agilent MassHunter Qualitative Analysis, version 10.0). The integration of all urinary molecular features was normalized to an internal standard (Cl-Tyr or NMS under positive and negative ion mode, respectively) and reported as their relative peak area (RPA). Temporal signal pattern recognition when using multiplexed separations in MSI-CE-MS [35] was used to reject spurious, background, and redundant (i.e., in-source fragments, isotopic signals, salt adducts, and same ion measured in both modes) molecular features when analyzing urine samples pooled from OO, EPA, and DHA sub-groups. Overall, 125 urinary metabolites satisfied the following inclusion criteria: (1) detected with adequate frequency (>60%) and technical precision (coefficient of variance, CV < 30%) after a repeat analysis of 33 QC runs comprised of pooled urine from all participants, and (2) detected with adequate frequency (>75%) in participant data measured at both time points (*n* = 176). This filtering approach resulted in a urine metabolome data matrix that excluded infrequently detected exogenous compounds (e.g., acetaminophen glucuronide, saccharin, etc.). Additionally, recovery standards were excluded from the urine metabolome data matrix (except F-Phe). Otherwise, urinary metabolites not detected in certain samples were replaced with half the value of the minimum response measured across the entire cohort if present below method detection limits, whereas putative missing data due to potential isobaric interferences were excluded from analysis. Lastly, the integrated response ratio for each metabolite to an internal standard or RPA was normalized to creatinine for each respective participant to correct for hydration status when relying on spot morning urine samples. Overall, urinary metabolites associated with the O3I were identified with high confidence (level 1) when spiked with authentic standards (if available), tentatively identified based on the annotation of MS/MS spectra with a consistent electrophoretic mobility (level 2), or were unidentified with an unknown chemical structure (level 3) if ion responses were too low to acquire MS/MS spectra without a suitable candidate reported in the HMDB. In all cases, urinary metabolites were annotated based on their characteristic accurate mass and relative migration time (*m*/*z*:RMT) under positive (p) or negative (n) ion mode together with their most likely molecular formula.

### 2.5. Statistical Analysis

All statistical analyses were conducted in R (v 4.1.2) [38] with R Studio (v 2022.7.2.576) [39]. R scripts are available upon request.

#### 2.5.1. Multivariate Analysis

Principal component analysis (PCA) was performed on the delta values of FAs, the O3I, and urine metabolites to compare the effect of OO, EPA, and DHA supplementation using the base-R stats function ‘princomp’ and ‘prcomp’ for fatty acid and metabolite data, respectively. Scaled PCA visualization plots were created with the package ‘factoextra’ (v 1.0.7) [40]. Partial least square–discriminant analysis (PLS-DA) was performed on metabolite variable delta values with the ‘mixOmics’ package (v 6.16.3) [41], where overall and balanced error rates (BER) were plotted to determine the optimal number of components for the final model. Sparse partial least square–discriminant analysis (sPLS-DA) was used to select for metabolites that responded to OO, EPA, or DHA supplementation.

#### 2.5.2. ANOVA Tests

Data normality and homogeneity of variance were assessed with the base-R Shapiro–Wilk test and Levene’s test from the ‘rstatix’ package (v 0.7.0.999) [42], respectively. For normal and non-normal data distributions, one-way analysis of variance (ANOVA) or Kruskal–Wallis tests were used to compare supplement groups at baseline, respectively. To determine the effects of EPA and DHA supplementation over time, creatinine-normalized urinary metabolite data were log_10_-transformed to establish normal distribution, and a linear mixed effects model (LMM) with random effects was fitted for each participant (‘nlme’ R package, v 3.1.157) [43] prior to two-factor, repeated-measures ANOVA analysis (‘stats’ R package, v 4.1.2) [38]. For metabolites with a significant group × time interaction effect (P_int_ < 0.05), a Bonferroni control was applied to within-factor *post hoc* pairwise t-tests using the ‘rstatix’ and ‘emmeans’ packages (v 1.4.8) [44]. For urinary metabolites not normally distributed after log_10_ transformation, a one-way Kruskal–Wallis test was performed at both time points followed by post hoc pairwise comparisons using Dunn’s test with Bonferroni adjustment (‘rstatix’ package). For all *post hoc* pairwise comparisons, *p*-adjusted values are reported. All analyses on erythrocyte FAs were conducted on relative % data; however, when possible, we also investigated quantitative (ng FA/mg Hb) data to confirm our findings.

#### 2.5.3. Integrated Data

Erythrocyte FA and urine metabolome datasets were integrated in a single dataset, with urinary metabolites as covariates and the O3I as the response variable. Isobaric or highly colinear urinary metabolites were removed prior to feature selection. Urinary metabolites in the integrated dataset were scaled from 0 to 10 to match the range in the O3I (the outcome variable). Feature selection was performed with a regularized LMM to select for relevant metabolites while including a random effect for participant. The LMM was implemented with the R package ‘glmmLasso’ (v 1.6.2) [45] with an L1 penalty. The tuning parameter lambda (λ) was selected through a grid search over a given range of λ values from 2000 to 0.0001, where the λ value with the lowest Bayesian information criterion (BIC) was selected for the final fitted model. All remaining metabolites in the final model with non-zero coefficients and *p* < 0.05 were identified. As a confirmation, feature selection was also conducted on a second integrated dataset with urine metabolites as covariates, but this time using quantitative EPA + DHA (ng FA/mg Hb) as the response variable, which was scaled from 0–700 to match the EPA + DHA (ng FA/mg Hb) range in the dataset. The diagnostic ability of selected creatinine-normalized urinary metabolites and their ratios were assessed using receiver operating characteristic (ROC) curves with the R package ‘pROC’ (v 1.18.0) [46], where optimal thresholds were selected via optimality criterion max(sensitivities + specificities), i.e., Youden’s J statistic.

#### 2.5.4. Correlation Plots

Pearson correlation coefficient correlation plots were generated with ‘ggplot2′ (v 3.3.5) [47] and ‘corrplot’ (v 0.92) [48] R packages. A correlation matrix comprised of FAs and urinary metabolites was calculated with the ‘psych’ (v 2.2.9) R package [49]. *p*-value adjustment was performed with the Holm–Bonferroni method.

## 3. Results

### 3.1. Patient Characteristics

**Figure 1:**
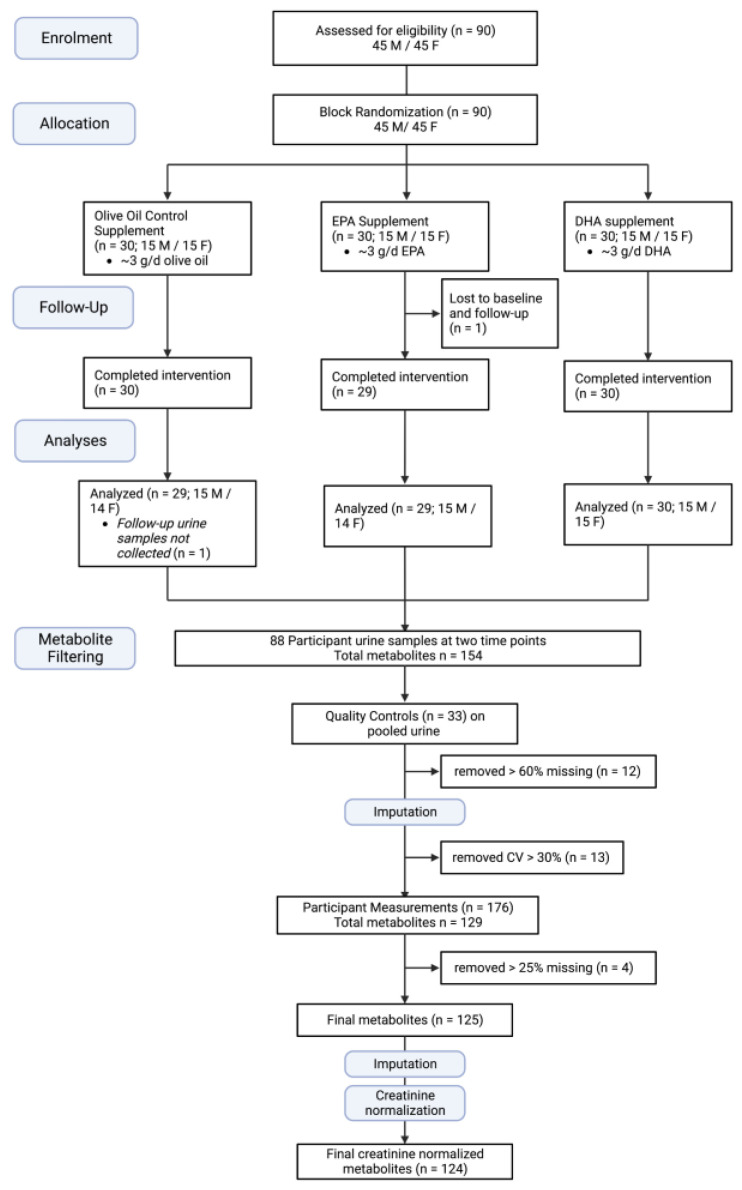
CONSORT flow diagram for the long-chain polyunsaturated omega-3 fatty acid randomized controlled trial. Urine metabolome quality assessments were performed on 33 repeat quality controls (QCs) of pooled urine and on participant data measured at both time points (*n* = 176), resulting in 124 creatinine-normalized urinary metabolites. Created with BioRender.com.

A total of 90 participants were enrolled into the study, where 30 (15 males and 15 females) were randomly allocated to each supplement group (Figure 1). Two participants did not provide final blood and urine samples, one in the EPA supplement group and one in the OO control group, the former of which did not provide all baseline samples/measurements. In total, data for 88 participants with matching blood and urine samples at both time points were used for the present analysis. 

### 3.2. Baseline Participant Data Analysis in the Three Supplement Groups

Group means for baseline participant data were calculated and are reported in Table 1. There were no significant differences between the three groups for baseline characteristics.

### 3.3. Blood Marker and Erythrocyte Fatty Acid Analyses

Fasted serum glucose, triglycerides, and cholesterol levels, as well as erythrocyte EPA, DHA, and the O3I, were measured at both time points and are presented in Table 2. No significant differences were observed between age, sex, and BMI-matched groups at baseline for any parameters.

As previously reported [50], fasted serum triglycerides were significantly reduced in the DHA supplement group. Interactions were observed for erythrocyte EPA and DHA levels, as well as the O3I, in the EPA and DHA supplementation groups. Specifically, individuals in the EPA group showed significant (*p* < 0.001) increases in %EPA (+3.4% ± 1.1) and the O3I (+3.0% ± 1.4), but non-significant decreases in %DHA (−0.5% ± 1.0). Individuals in the DHA group showed significant (*p* < 0.001) increases in %EPA (+0.7% ± 0.8), %DHA (+4.2% ± 1.2), and the O3I (+4.9% ± 1.3), which was similarly reflected in the quantitative FA data. As expected, no differences for erythrocyte EPA, DHA, or the O3I were observed in the OO control group.

### 3.4. Identification of Urinary Metabolites Associated with EPA and/or DHA Supplementation

To avoid spurious results, we applied multiple statistical methods to identify urinary metabolites associated with the O3I. These urinary metabolites comprised 17 (out of 125) annotated unknown ions via high-resolution MS tentatively identified using collision-induced dissociation MS/MS (Table S1). First, we used supervised and unsupervised clustering methods to obtain a general overview of the urine metabolome data variance that distinguishes the supplement groups from one another. Second, we used pairwise analyses to examine changes in creatinine-normalized metabolite responses in relation to supplementation and time. Finally, we investigated those urinary metabolites that directly contributed to predicting variability in the O3I. Collectively, these complimentary statistical approaches were used to select and identify urinary metabolites that were consistently and temporally associated with n3-LCPUFA status as compared to the OO control.

#### 3.4.1. Distinguishing Supplementation Groups with Unsupervised and Supervised Clustering Approaches

First, we performed a global analysis to examine if urinary metabolite profiles could distinguish between the three supplement groups from baseline. Overall, the technical variance for repeated analysis of 125 metabolites (including creatinine) in a pooled urine sample was acceptable with a median CV = 13.4% for QCs (*n* = 33) as compared to their greater between-subject biological variance with a median CV = 60.5% (*n* = 178). Two dimensional PCA plots of 124 creatinine-normalized urinary metabolites at both baseline (Figure S1a) and 12 weeks (Figure S1b) were unable to distinguish the three supplement groups from one another. Furthermore, a PCA plot corresponding to the delta response values (i.e., RPA after supplementation–RPA at baseline) for urinary metabolites was also unable to distinguish the three supplement groups (Figure S1c), with the first two components (PC1 and PC2) only explaining 20.4% of the total variance in the dataset. Similarly, the three supplement groups were not well distinguished by relative %FA (Figure S1d) or by quantitative FA data (Figure S1e). In all instances, participants from all groups were clustered around the centre of each plot with no clear distinction observed between groups.

**Figure 2:**
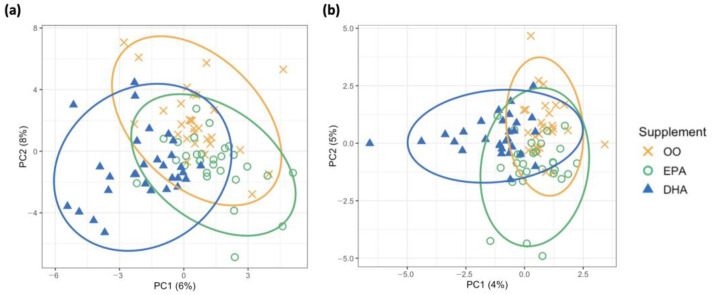
Participant plots of relative peak area (RPA) delta values for (**a**) 124 creatinine-normalized metabolite responses in PLS-DA and (**b**) 15 selected urinary metabolites for each principal component in sPLS-DA, coloured by group (OO = orange, EPA = green, DHA = blue).

We next performed supervised PLS-DA and sparse PLS-DA (sPLS-DA) to better distinguish the three supplementation groups in this study (Figure 2). While less total variation was explained by the first two principal components (i.e., PC1 + PC2 = 14% and 9% for Figure 2a,b, respectively) than in the PCA model, the visual separation between groups was slightly improved. Since we hypothesized that only a small subset of urinary metabolites would relate to changes in EPA and/or DHA erythrocyte content, we next performed a sPLS-DA for urinary metabolite biomarker selection.

**Figure 3:**
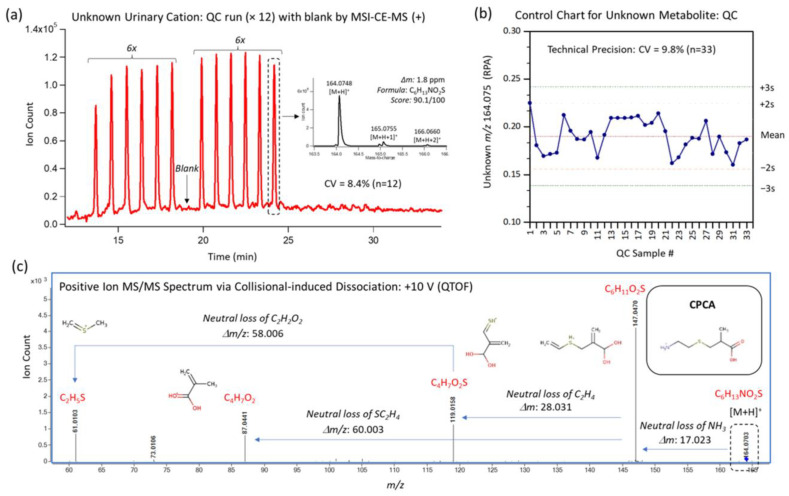
Structural elucidation of *S*-carboxypropylcysteamine (CPCA). (**a**) Representative extracted ion electropherogram when using MSI-CE-MS under positive ion mode for CPCA, highlighting a serial injection of 12 urine samples and a blank in a single run. (**b**) A control chart for urinary CPCA based on the repeated analysis of pooled urine as quality control (QC) samples demonstrating acceptable technical precision (CV = 9.8%, *n* = 33). (**c**) Annotated MS/MS spectrum under an optimal collisional energy depicting the formation of four characteristic fragment ions and their putative structures.

Variable selection via sPLS-DA identified the top-ranked 15 urinary metabolites associated with OO, EPA, and DHA supplementation for both PC1 and PC2 (Figure S2). The metabolites selected in PC1 were related to DHA supplementation (blue), whereas those selected in PC2 largely corresponded to metabolites related to EPA supplementation (green). The top-ranked urinary metabolites having the largest weighting on PC1 were an unknown divalent anion (221.075:0.927:n), as well as an unknown cation (164.074:0.614:p) that was tentatively identified as *S*-carboxypropylcysteamine (CPCA) based on annotation of its MS/MS spectrum at an optimal collisional energy under positive ion mode (Figure 3) together with its co-migration after spiking a standard of CPCA in pooled urine (Figure S3). Other isobaric candidate ions having the same molecular formula (e.g., ethione, *N*-methylmethionine, *S*-methylmethioine, etc.) were excluded as likely candidates based on their slower positive mobility (at pH 1.8) due to a more acidic alpha-carboxylic acid moiety, resulting in their longer apparent migration times (e.g., *S*-propylcysteine). Overall, urinary CPCA was measured consistently with acceptable technical precision (CV = 9.8%, *n* = 33) throughout the study. The third and fourth most significant urinary metabolites classified via sPLS-DA along PC1 were tetrahydroaldosterone glucuronide and an unknown anion isomer (241.120:0.653:n), which was tentatively identified as a dipeptide when using MS/MS in negative ion mode, namely pyroglutamylisoleucine (pGlu-Ile) (Figure S4). As there were two partially resolved isobars in the extracted ion electropherogram with analogous MS/MS spectra, isobars were distinguished by their apparent migration times, with the slower migrating isomer likely being pyroglutamylleucine (pGlu-Leu). Overall, the urinary metabolites contributing the most loading weight for PC1 (Figure S2a) were an unknown dianion (221.075:0.927:n), CPCA (164.074:0.613:p)*,* tetrahydroaldosterone glucuronide, and pGlu-Ile. Top loading weights for PC2 (Figure S2b) corresponded to choline, glucuronic acid, an unknown dianion (88.004:1.622:n), and quinic acid. Structural elucidation of the unknown dianion (221.075:0.927:n) was not feasible due to its low abundance in urine that prevented the acquisition of a MS/MS spectra (Figure S5).

#### 3.4.2. Individual Urinary Metabolite Changes in Response to Supplementation over Time

**Figure 4:**
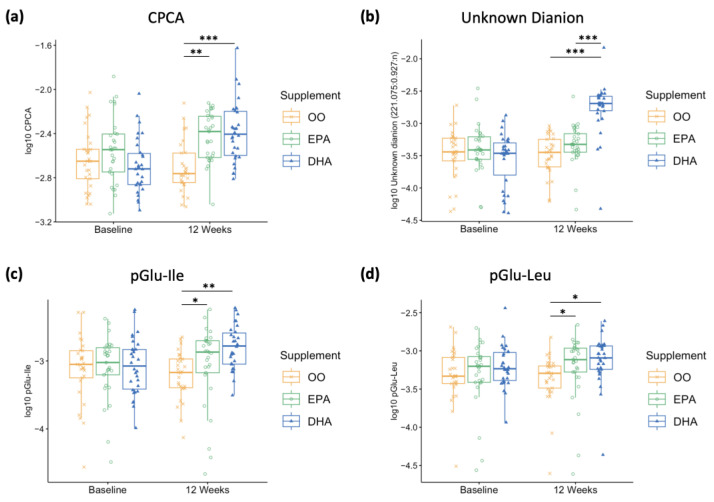
Creatinine-normalized urinary metabolite responses after log_10_ transformation for (**a**) *S*-carboxypropylcysteamine (CPCA, 164.074:0.613:p), (**b**) an unknown dianion (221.075:0.927:n), and (**c**,**d**) a dipeptide tentatively identified as pGlu-Ile and pGlu-Leu, respectively, at baseline and final (12 weeks) for olive oil (OO, yellow), EPA (green), and DHA (blue). Mean group differences were analyzed with 2-factor repeated measures ANOVA for CPCA and Kruskal–Wallis test for the others. Post hoc Bonferroni-adjusted results between each supplement group are reported, (*) *p* < 0.05, (**) *p* < 0.001 and (***) *p* < 0.0001. OO = olive oil, EPA = eicosapentaenoic acid, DHA = docosahexaenoic acid, pGlu-Ile = pyroglutamylisoleucine, pGlu-Leu = pyroglutamylleucine.

We next examined changes in individual urinary metabolites in response to supplementation and time. Most (88 out of 124) urinary metabolites were normally distributed after log_10_ transformation, while 36 were non-normally distributed. For normally distributed metabolites, significant interactions (P_int_ < 0.05) were observed for nine urinary metabolites, of which four remained significant after applying a Bonferroni adjustment: CPCA (*p* < 0.001), tiglylglycine (*p* < 0.001), an unknown cation (300.215:0.841:p, *p* = 0.004), and glucuronic acid (*p* = 0.014). *Post hoc* analyses found no differences in CPCA at baseline between the three groups; however, the excretion of creatinine-normalized CPCA increased significantly following EPA and DHA supplementation compared to OO (EPA vs. OO, *p* < 0.001; DHA vs. OO, *p* < 0.001) (Figure 4a). Additionally, DHA supplementation significantly increased the amount of CPCA excreted in urine (baseline vs. 12 weeks, *p* < 0.001). *Post hoc* analysis also revealed significant differences between groups for tiglylglycine at baseline (OO vs. EPA, *p* = 0.004; EPA vs. DHA, *p* = 0.016) and after supplementation (OO vs. DHA, *p* = 0.012), where there was a significant decrease with DHA supplementation (*p* < 0.001). No group differences were found for the other two urinary metabolites.

For non-normally distributed metabolites, we also identified significant group differences at 12 weeks for an unknown dianion (221.075:0.927:n) (*p* < 0.001), pGlu-Ile (*p* < 0.001), and pGlu-Leu (*p* = 0.002). *Post hoc* analyses found significant group differences at 12 weeks for the unknown dianion (221.075:0.927:n) (OO vs. DHA, *p* < 0.001; EPA vs. DHA, *p* < 0.001) with a significant increase after DHA supplementation (*p* < 0.001) (Figure 4b). Both pGlu-Ile and pGlu-Leu have significant differences at 12 weeks between OO and EPA (*p* = 0.046 and *p* = 0.015, respectively) and OO vs. DHA (*p* < 0.001 and *p* = 0.002, respectively) (Figure 4c,d). Another urinary metabolite, an unknown cation (201.160:0.644:p), was identified as having group differences at baseline, and *post hoc* analyses revealed a significant difference between OO and EPA (*p* = 0.002). No significant group differences were found for any other non-normally distributed urinary metabolite.

#### 3.4.3. Urinary Metabolites Associated with the O3I

Next, we examined if any urinary metabolites were highly associated with the O3I. Because we hypothesized that only a small subset of urinary metabolites would be associated with changes in the O3I, we performed LASSO regression to identify those metabolites that were highly linked to changes in the O3I. The results of selecting a subset of urinary metabolites associated with the O3I response are reported in Table S2. In total, four urinary metabolites remained in the final fitted model. All four urinary metabolites were identified in a complementary analysis performed on an integrated dataset using summed EPA + DHA (ng/mg Hb) as the response variable, which also identified an additional three urinary metabolites (Table S2). The estimated effects of the seven selected urinary metabolites on the O3I and/or EPA + DHA (ng/mg Hb) are depicted in Figure S6. Of the selected urinary metabolites in both datasets, the unknown dianion (221.075:0.927:n), CPCA, and tetrahydroaldosterone glucuronide had positive estimated effects, i.e., higher concentrations in urine were associated with increases in the erythrocyte O3I and/or EPA + DHA (ng/mg Hb). In contrast, an unknown cation (188.175:0.259:p; first migrating isobar), tentatively identified as *N1*-acetylspermidine (*N1*-AcSpm; Figure S7), had a negative estimate effect in both datasets, i.e., a reduction in its concentration was associated with an increase in the O3I and/or EPA + DHA (ng/mg Hb).

Collectively, these three statistical approaches identified 17 urinary metabolites significantly associated with n3-LCPUFA or the O3I (Table 3).

### 3.5. Urinary Metabolites Predict Supplement Groups and the O3I

The 17 urinary metabolites identified across these three analyses were then examined for their ability to act as a classifier for EPA and/or DHA supplementation, as well as a classifier for low (<4%) and high (>8%) O3I, using ROC curves. 

**Figure 5:**
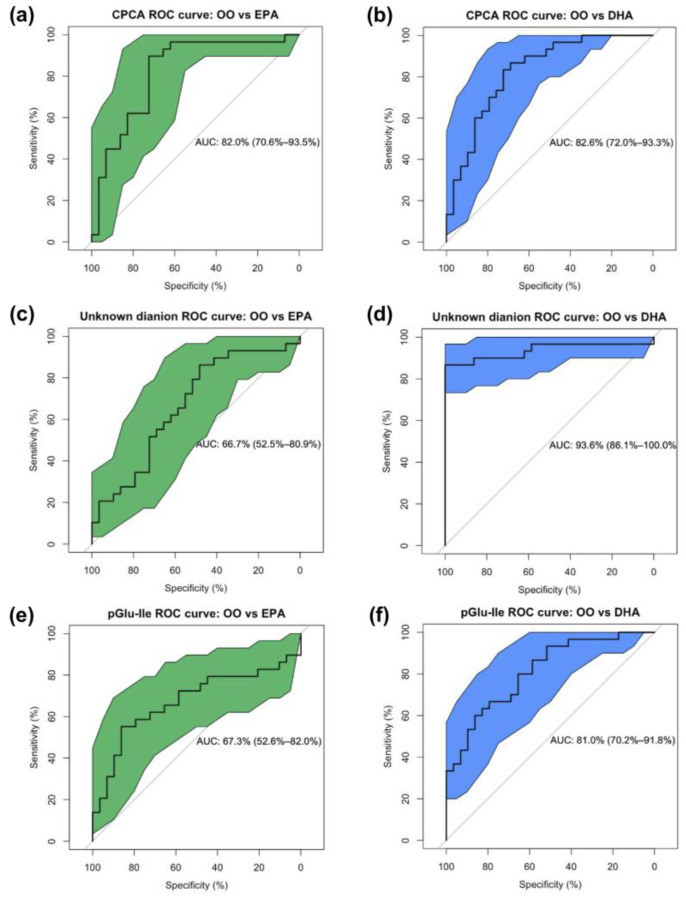
ROC curves of the sensitivity and specificity of creatinine-normalized urinary metabolites to classify OO vs. EPA (confidence interval in green) and OO vs. DHA (confidence interval in blue) at 12 weeks; (**a**) CPCA AUC = 82.0% and (**b**) 82.6%; (**c**) an unknown dianion (221.075:0.927:n) AUC = 66.7% and (**d**) 93.6%; and (**e**) pGlu-Ile AUC = 67.3% and (**f**) 81.0%, respectively. CPCA = *S*-carboxypropylcysteamine, pGlu-Ile = pyroglutamylisoleucine.

As a supplement group classifier between OO and EPA, or OO and DHA, at 12 weeks, urinary CPCA consistently outperformed the other top 16 metabolites in both EPA and DHA supplement groups with an AUC of 82.0% (sensitivity = 89.6%, specificity = 72.4%) and 82.6% (sensitivity = 83.3%, specificity = 72.4%), respectively (Figure 5a,b). The unknown dianion (221.075:0.927:n) outperformed all other metabolites for the OO versus DHA comparison only (AUC = 93.6%, sensitivity = 86.7%, specificity = 100.0%), but had a poor overall performance for differentiating EPA and OO supplementation (AUC = 66.7%) (Figure 5c,d). Apart from urinary isobars pGlu-Ile and pGlu-Leu that had AUCs of 67.3% and 70.9% for OO versus EPA and 81.0% and 75.9% for OO versus DHA, respectively, the other positively correlated metabolites to the O3I generated lower AUCs ranging from 54.7% to 68.1% (Figure 5e,f and Table 3). The combined performance of CPCA and the unknown dianion (221.075:0.927:n) showed a slight improvement over CPCA alone in distinguishing EPA supplementation from OO (AUC = 83.0%) but resulted in a substantial improvement in differentiating DHA from OO (AUC = 92.4%).

For urinary metabolites inversely correlated with the O3I, tiglylglycine had an AUC of 69.4% and 71.3% for OO versus EPA or DHA supplementation, respectively, while AUC values for *N1*-AcSpm and carboxybutylhomocysteine were ~50–60% except for OO versus EPA for the *N1*-AcSpm (AUC = 70.5%). For urinary metabolites that were identified in at least one of the three statistical analyses, but showed no significant association with n3-LCPUFA supplementation or the O3I, AUC values were all less than 70%. 

Ratiometric combinations showed the highest AUC in EPA versus OO (AUC = 86.1%) to be a ratio of CPCA and *N1*-AcSpm and the highest AUC in DHA versus OO (AUC = 95.6%) to be a ratio of an unknown dianion (221.075:0.927:n) and tiglylglycine. Importantly, supplement group distinguishment for the unknown dianion (221.075:0.927:n), CPCA, pGlu-Ile, and pGlu-Leu remained similar when not normalizing metabolite responses to creatinine, suggesting a robustness in predictor ability even without correction for hydration status in this cohort of young Canadian adults.

When classifying O3I status, both urinary CPCA and the unknown dianion (221.075:0.927:n) outperformed all other metabolites when distinguishing between an O3I of <4% and >8% (Table 3). In this case, the top performing classifier was the unknown dianion (221.075:0.927:n) with an AUC of 89.4% (sensitivity = 83.3%, specificity = 94.4%), while CPCA was the second best classifier with an AUC of 73.1% (sensitivity = 83.3%, specificity = 57.3%).

### 3.6. Increases in Urinary CPCA Are Associated with n3-LCPUFA Erythrocyte Content 

**Figure 6:**
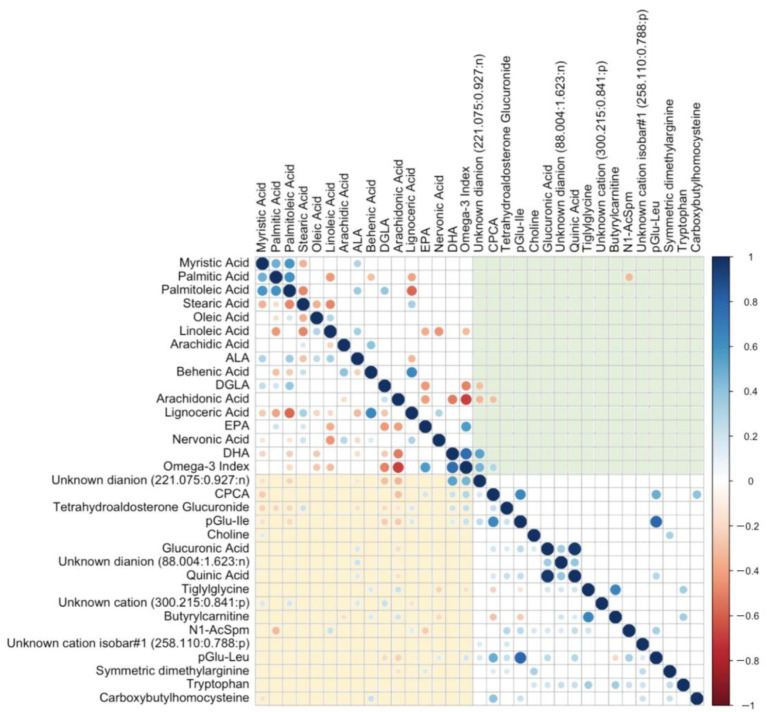
Correlation plot of relative % fatty acids in erythrocytes and top 17 selected metabolites, where significant (*p* < 0.05 below diagonal, P_adj_ < 0.05 above diagonal) correlations are indicated by sphere size (strength) and colour (blue = positive, red = negative). Nonsignificant correlations are depicted by blank cells. Urinary metabolites with significant un-adjusted correlations to erythrocyte fatty acids are depicted in the bottom left quadrant (yellow); urinary metabolites with significant adjusted correlations to erythrocyte fatty acids are depicted in the top right quadrant (green). ALA = α-linolenic acid, DGLA = di-homo-γ-linolenic acid, EPA = eicosapentaenoic acid, DHA = docosahexaenoic acid, CPCA = *S*-carboxypropylcysteamine, pGlu-Ile = pyroglutamylisoleucine, pGlu-Leu = pyroglutamylleucine, *N1*-AcSpm = *N1*-Acetylspermidine.

Finally, we performed additional analyses on the two lead urinary biomarkers of the O3I (CPCA and the unknown dianion 221.075:0.927:n) that were systematically identified in all three statistical approaches (Table 3). Specifically, we examined whether the changes observed in these urinary metabolites were specific to changes in the O3I through measuring Pearson correlation coefficients (*r*) with all other measured erythrocyte fatty acids (Figure 6). Urinary CPCA was positively correlated with the O3I (*r* = 0.30, *p* < 0.001) but also showed correlations with both EPA (*r* = 0.19, *p* = 0.012) and DHA (*r* = 0.21, *p* = 0.004) individually. CPCA was also inversely correlated with AA (*r* = −0.30, *p* < 0.001) and myristic acid (*r* = −0.26, *p* < 0.001). However, CPCA was not correlated with any other measured fatty acid. The unknown dianion (221.075:0.927:n) was more strongly correlated with the O3I (*r* = 0.47, *p* < 0.001) and with DHA (*r* = 0.53, *p* < 0.001) but was not correlated with EPA. Also, this acidic urinary metabolite was negatively correlated with di-homo-γ-linolenic acid (DGLA) (*r* = −0.30, *p* < 0.001) and arachidonic acid (AA) (*r* = −0.33, *p* < 0.001). 

These associations between CPCA and the unknown dianion (221.075:0.927:n) with relative % erythrocyte fatty acids were further confirmed when measuring correlations with quantitative erythrocyte fatty acid data as reported as their absolute amounts normalized to total hemoglobin (ng FA/mg Hb). However, only two associations remained significant after adjustment for multiple testing for the unknown dianion (221.075:0.927:n) (DHA, *r* = 0.50, P_adj_ < 0.001 and sum of EPA + DHA, *r* = 0.42, P_adj_ < 0.001), yet none were significant after adjustment in the case for CPCA. Correlation plots between the unknown dianion (221.075:0.927:n) and CPCA and the O3I and sum of EPA + DHA (ng FA/mg Hb) are shown in Figure S8.

Since both CPCA and the unknown dianion (221.075:0.927:n) were found to be correlated with the O3I (positively) and AA (inversely), and that the O3I and AA were themselves strongly and inversely correlated with one another (*r* = −0.69), we also examined whether an O3I/AA ratio would result in improved correlation coefficients with these two urinary metabolites than O3I alone. We found positive correlations between the unknown dianion (221.075:0.927:n) and CPCA with the O3I/AA ratio (*r* = 0.47, *p* < 0.001; *r* = 0.32, *p* < 0.001, respectively), which are similar to the correlations between these respective urinary metabolites and the O3I alone.

Urinary peptide isomers, pGlu-Ile and pGlu-Leu were also found to be positively correlated with the O3I (*r* = 0.22 and *r* = 0.19, respectively) with a corresponding negative association with AA (*r* = −0.26 and *r* = −0.23, respectively). We also found urinary tiglylglycine to be positively correlated with behenic acid (*r* = 0.16, *p* = 0.028) and AA (*r* = 0.22, *p* = 0.003), and inversely correlated with nervonic acid (*r* = −0.22, *p* = 0.003) and the O3I (*r* = −0.19, *p* = 0.019). However, none of these associations remained significant after adjusting for multiple testing. *N1*-AcSpm was positively correlated with linoleic acid and lignoceric acid and negatively correlated with EPA and palmitic acid, where only the latter remained significant after adjustment for multiple testing (*r* = −0.33, P_adj_ = 0.004).

## 4. Discussion

To the best of our knowledge, this study is the first to investigate urinary metabolites as putative non-invasive biomarkers of the O3I using a non-targeted metabolomics approach. Our results identified urinary CPCA and an unknown dianion (221.075:0.927:n) as novel candidate urinary biomarkers for O3I status in healthy, young individuals. While the unknown dianion (221.075:0.927:n) was also identified as an indicator of DHA supplementation specifically, CPCA was found to be associated with both EPA and DHA supplementation as compared to OO. More specifically, both candidate urinary metabolites were positively associated with the O3I, and when compared to all 15 measured erythrocyte membrane-derived phospholipid fatty acids, creatinine-normalized urinary CPCA levels showed specificity in their association with the O3I, whereas the unknown dianion (221.075:0.927:n) showed a greater association with DHA. As a supplement group classifier between EPA or DHA relative to OO, urinary CPCA consistently outperformed other selected urinary metabolites when using ROC curves (AUC > 80%), whereas the unknown dianion showed the highest performance specifically for DHA (AUC > 90%) with poor sensitivity for EPA. Similarly, both CPCA (AUC = 73%) and the unknown dianion (AUC = 89%) outperformed other urinary metabolites when classifying a low (<4%) versus high (>8%) O3I. Taken together, our robust analyses have positioned urinary CPCA and the unknown dianion (221.075:0.927:n) as novel surrogates for the O3I. Collectively, this work lays the foundation for developing a convenient and non-invasive approach to assess an individual’s n3-LCPUFA status.

Urinary biomarkers represent an attractive option for nutrient monitoring. Traditional dietary assessments estimate nutrient status, but are limited in their ability to accurately capture variation in food quality, intake, absorption, and metabolism [51]. Therefore, biomarkers have the advantage of more accurately indicating the amount of a nutrient available to tissues and organs. Current validated nutrient biomarkers of n3-LCPUFA status such as the O3I [52] require a small blood sample. Recently, Ly et al. [53] reported two phosphatidylcholine species can serve as circulating surrogate biomarkers of the O3I directly in serum or plasma rather than hydrolyzed EPA and DHA measured from the phospholipid fraction of erythrocyte membranes. Yet, access to blood specimens may still represent a barrier to their use at both the individual and population levels. In contrast, the use of urinary metabolites as a proxy for the O3I could improve ease of monitoring while capturing physiologically relevant information. Although numerous reports in the literature have identified potential candidate biomarkers of n3-LCPUFA status, very few have been validated and are therefore currently of limited use. While a validation pipeline for biomarkers of food intake and/or nutrient status has been proposed [54], meeting all criteria for establishing the biological validity and analytical performance of a biomarker remains a challenge. Nevertheless, the power of a validated urinary biomarker for nutrient status has wide-scale implications for use in personalized health, nutrition and epidemiological research, and clinical risk assessment for cardiovascular events and hypertriglyceridemia, amongst others. 

Through our analyses, we found that urinary CPCA was significantly correlated with changes in the O3I and AA satisfying both a dose and temporal response as criteria. Moreover, urinary CPCA was reliably measured with acceptable technical precision (mean CV < 10%) with no missing values using a validated multiplexed separation platform and data workflow for metabolomics based on MSI-CE-MS [29]. Also, urinary CPCA increased consistently following both EPA and DHA supplementation in our cohort of young Canadian adults having a low average O3I status at baseline. Overall, no other putative urinary biomarker associated with the O3I identified in our study had higher sensitivity (89.6% and 83.3%) and specificity (72.4% and 72.4%) for both EPA and DHA supplementation, respectively. We hypothesized that the inverse relationship between CPCA and AA stems primarily from the known reduction in AA membrane content observed with increased n3-LCPUFA intake [1]. Indeed, we found that when the relative levels of erythrocyte EPA and DHA increased, there were proportional reductions in AA. In a dose–response randomized controlled trial examining EPA + DHA supplementation in healthy male and female participants, Flock et al. [52] reported that erythrocyte EPA and DHA content increased concomitant with comparable reductions in AA content. Similarly, Vidgren et al. [55] showed significant reductions in erythrocyte AA content in individuals consuming a fish oil supplement or eating a fish diet. Our findings align with these previous studies. Due to the close relationship between n3-LCPUFA and AA erythrocyte membrane content, the specificity of CPCA related to the O3I may falsely implicate the latter. Indeed, there was an equivalent strength of association of urinary CPCA inversely with AA and positively with the O3I (*r* = 0.30) as compared to EPA or DHA alone. Nevertheless, increases in the O3I concomitant with decreases in AA are generally indicative of good health [56].

Urinary metabolites related to fish intake have been proposed as potential urinary biomarkers in previous studies. In a cross-sectional analysis of the INTERMAP study, Gibson et al. [23] found TMAO and taurine were capable of distinguishing between low and high fish intake in a Japanese subpopulation. Our study identified and quantified urinary TMAO, but we did not find that this metabolite was capable of distinguishing between low and high O3I levels. Taken together, this suggests that TMAO may be a urinary biomarker related to marine meat protein intake rather than n3-LCPUFA. A randomized controlled trial in individuals with type-2 diabetes supplemented with fish oil, flaxseed oil, or corn oil showed significant increases in urinary CMPF in the fish oil group, which was also highly correlated to serum CMPF [24]. The association of urinary CMPF could not be evaluated in our study as it was not detected via MSI-CE-MS. Therefore, both TMAO and CMPF require further evaluation to clarify their use as biomarkers of fish/fish oil intake versus n3-LCPUFA status.

To the best of our knowledge, only two previous studies have explored associations between the O3I and urinary metabolites. Bigornia et al. [22] reported an inverse relationship between the O3I and depressive symptoms in older adults with obesity having high urinary 8-OHdG, i.e., a biomarker of oxidative stress. However, no association was found between the O3I and urinary 8-OHdG at baseline and it was not evaluated at the 2 y follow-up, which suggest that this relationship between depressive symptoms and the O3I may be indirectly related to oxidative stress. In a separate cross-sectional study in young and healthy adults, Filipovic et al. [21] reported an inverse relationship between the O3I and the albumin-to-creatine (ACR) ratio after adjusting for various covariates, such as age, sex, BMI, and smoking status. Neither 8-OHdG nor albumin were detected in our urinary metabolomic analysis when using MSI-CE-MS, which prevents our independent verification of the relationship between these metabolites and the O3I in our study.

Currently, there are nine isobaric candidate metabolites in the HMDB that match the most likely molecular formula for the urinary metabolite we have tentatively identified as CPCA (164.074:0.613:p-C_6_H_13_NO_2_S). While the unambiguous identification of CPCA is ongoing, we have ruled out four isobaric ions based on migration behaviour and structural properties that impact their ionization state (i.e., pKa) and corresponding migration times. Of the remaining isobaric ion candidates reported in the HMDB, the structural properties of CPCA’s functional groups best reflect the migration time and characterization shown in the extracted ion electropherogram (Figure 3). Furthermore, additional studies are necessary to clarify the underlying biochemical link(s) between n3-LCPUFA and CPCA as well as establish reference intervals for urinary CPCA in larger populations to define optimal cut-off concentrations associated with the O3I.

Additionally, while demonstrating a strong association to the O3I, an unknown dianion (221.075:0.927:n) showed specificity in its relationship to DHA supplementation with seemingly little-to-no association with EPA supplementation. In the absence of a confirmed identification for this urinary metabolite, it is challenging to speculate on the biochemical significance of this finding and its link with DHA; however, this currently unidentified metabolite may prove to be a specific marker of DHA in the body and thus warrants continued investigation. Improved concentration sensitivity is needed to measure this lower abundance hydrophilic urinary metabolite, which is also needed when acquiring better quality MS/MS spectra.

Our study has several strengths. First, the participant samples used were collected during a randomized placebo-controlled trial that included matching baseline and 12-week fasted blood and first-void urine samples. Second, erythrocyte fatty acid measurements were examined using both relative % and quantitative (ng FA/mg Hb) values, thus allowing for the robust verification of the relationship between erythrocyte EPA and DHA with CPCA. All three statistical methods performed on all 124 creatinine-normalized urinary metabolites confirmed similar results, where CPCA and an unknown dianion (221.075:0.927:n) were consistently identified using several different approaches. Additionally, we conducted an initial proof-of-principle study 2 years prior to the present full study that first identified a relationship between CPCA and high-dose DHA intake relative to OO with good mutual agreement and reproducibility, thus demonstrating the stability of CPCA in frozen urine samples following repeat analysis after thawing and long-term storage. Finally, to rule out potential metabolite contamination, we analyzed both lipid and aqueous extracts of the OO, EPA, and DHA capsules and did not detect CPCA, the unknown dianion (221.075:0.927:n), and other polar/hydrophilic biomarker candidates reported in this work, reinforcing that the changes in concentration of these endogenous urinary metabolites reflect biochemical changes in study participants following high-dose EPA and/or DHA intake.

There are also several limitations to our study. First, we acknowledge the small and relatively homogenous sample size of the current study. Indeed, participants were all young (18–30 y) and healthy individuals that do not necessarily reflect variation seen in free-living populations. Thus, the generalizability of urinary CPCA and the unknown dianion (221.075:0.927:n) as non-invasive biomarkers for the O3I needs to be confirmed in populations that vary in age, ethnicity, genetics, and health status. Additionally, other lifestyle factors were not accounted for during the clinical trial, such as physical activity, alcohol consumption, and dietary patterns. Future studies will need to measure macro- and micronutrient intakes to ensure that changes in urinary metabolites are specific to changes in n3-LCPUFA intake. Furthermore, first-void urine samples used in our metabolomics analyses are more susceptible to acute or diurnal changes in urine composition, which may diminish the accuracy of associations drawn between urine metabolites and long-term changes in erythrocyte fatty acid composition. As such, 24 h urine samples and/or several repeat spot urine samples collected over time from the same individual could provide a more reliable metabolite assessment of O3I status and improve the strength of associations between urinary metabolites and erythrocyte fatty acid composition. Future work needs to explore the stability of these metabolites in urine through comparing fasting first-void urine samples with 24 h samples, as well as day-to-day variability. Moreover, urine samples were analyzed on a single metabolomic platform (MSI-CE-MS), which is well suited for identifying polar or ionic compounds since they are predominantly excreted in human urine [57]. However, due to the selectivity and sensitivity of liquid chromatography, gas chromatography, and CE-MS techniques to capture different types of compounds, the use of complementary instrumental platforms may uncover additional urinary biomarkers of the O3I [58,59]. Ultimately, this could lead to the identification of a larger panel of urinary metabolites that may reflect changes in the O3I compared to CPCA and/or the unknown dianion (221.075:0.927:n) alone while also independently validating biomarker outcomes across platforms [60]. Furthermore, it is unclear what minimum dosage of n3-LCPUFA is needed to elicit a significant increase in these candidate urinary biomarkers above endogenous background levels in human urine. Since participants in the present trial received moderately high doses of EPA or DHA (~3 g per day), future studies should consider different doses and formulations of n3-LCPUFA supplementation. Finally, the underlying biochemical connection between these candidate urinary biomarkers and n3-LCPUFA remains to be elucidated. 

## 5. Conclusions

We report the identification of a novel panel of urinary metabolites as robust, non-invasive biomarkers of n3-LCPUFA status. Specifically, our findings reveal that urinary CPCA and an unknown dianion (221.075:0.927:n) may serve as promising biomarkers for the O3I, and thereby lay the groundwork for establishing an objective and non-invasive test for the personalized assessment of n3-LCPUFA status. We anticipate that an eventual non-invasive test could be used for early screening of health risks and self-monitoring of the O3I following personalized dietary changes. However, additional studies confirming the sensitivity and specificity of these urinary metabolites as biomarkers of the O3I require further validation prior to considering their clinical utility.

## Figures and Tables

**Table 1:** Participant baseline characteristics ^1^.

	OO	EPA	DHA	*p*-Value
M/F, *n*	15/15	15/14	15/15	
Age, y	21.1 ± 1.9	21.4 ± 2.2	22.2 ± 2.3	0.184
Weight, kg	69.0 ± 10.9	71.0 ± 12.0	72.7 ± 14.9	0.616
BMI, kg/m^2^	24.1 ± 3.5	23.1 ± 2.8	23.7 ± 3.4	0.613

^1^ Values expressed as mean ± SD. *p*-values were determined using one-way ANOVA. n for number of participants, OO = olive oil, EPA = eicosapentaenoic acid, DHA = docosahexaenoic acid.

**Table 2:** Blood measurements at baseline and after olive oil, EPA, and DHA supplementation for 12 weeks ^1^.

	OO (*n* = 30)		EPA (*n* = 29)		DHA (*n* = 30)		ANOVA		
	Baseline	Final	Baseline	Final	Baseline	Final	P_int_	P_treatment_	P_time_
Glucose, mmol/L	4.6 ± 0.3	4.7 ± 0.4	4.8 ± 0.3	4.9 ± 0.3	4.7 ± 0.3	4.9 ± 0.3	0.942	0.006	<0.001
Triglycerides, mmol/L	0.9 ± 0.4	0.9 ± 0.4 ^a,c^	0.8 ± 0.3	0.8 ± 0.3 ^a,b^	0.8 ± 0.2	0.6 ± 0.1 ^b,$^	0.049	0.023	0.028
Cholesterol, mmol/L	4.4 ± 0.9	4.6 ± 0.9	4.5 ± 0.7	4.5 ± 0.7	4.3 ± 0.8	4.5 ± 0.9	0.400	0.809	0.108
EPA, ng FA/mg Hb	27.6 ± 9.4	30.6 ± 11.3 ^c^	27.9 ± 10.7	225.5 ± 60.2 ^a,$^	25.9 ± 10.0	68.6 ± 25.2 ^b,$^	<0.001	<0.001	<0.001
DHA, ng FA/mg Hb	176.4 ± 40.8	172.8 ± 40.6 ^b^	161.4 ± 39.6	148.5 ± 43.6 ^b^	150.4 ± 32.7	401.8 ± 76.3 ^a,$^	<0.001	<0.001	<0.001
EPA, relative %	0.5 ± 0.2	0.5 ± 0.2 ^c^	0.5 ± 0.2	3.9 ± 1.1 ^a,$^	0.5 ± 0.2	1.2 ± 0.4 ^b,$^	<0.001	<0.001	<0.001
DHA, relative %	3.2 ± 0.7	3.0 ± 0.5 ^b^	3.0 ± 0.6	2.5 ± 0.5 ^b^	2.9 ± 0.5	7.1 ± 1.0 ^a,$^	<0.001	<0.001	<0.001
O3I	3.7 ± 0.8	3.5 ± 0.6 ^c^	3.5 ± 0.7	6.5 ± 1.2 ^b$^	3.4 ± 0.6	8.3 ± 1.2 ^a$^	<0.001	<0.001	<0.001

^1^ Values expressed as mean ± SD. Blood and FA variables were analyzed using 2-way repeated measures ANOVA to examine the main effects of supplement group and time (P_treatment_ and P_time_) as well as the interaction effects (P_int_ = Treatment × Time). Post hoc pairwise comparison results for variable measurements that are significantly different are reported with different superscript letters (across time) and with $ (within supplement group). OO = olive oil, EPA = eicosapentaenoic acid, DHA = docosahexaenoic acid, O3I = Omega-3 Index.

**Table 3:** Significant urinary metabolites associated with n3-LCPUFA supplementation or O3I status identified using three statistical tests.

	Test	sPLS-DA	ANOVA			LASSO		ROC Curves Group at 12 Weeks		ROC Curves Low vs. High O3I ^2^
	Outcome	Supplement Group (Δ)	Supplement Group × Time	Pairwise (vs. OO) at 12 Weeks	Pairwise (Change vs. Baseline)	O3I	EPA + DHA	AUC (%)	AUC (%)	AUC (%)
Metabolite ^1^	*m*/*z*:RMT:mode		P_int_					OO vs. EPA	OO vs. DHA	<4% vs. >8% O3I
Unknown dianion [M − 2H]^2−^	221.075:0.927:n	DHA	<0.001 *	↑DHA	↑DHA	↑	↑	66.7	93.6	89.4
*S*-Carboxypropylcysteamine (CPCA)	164.074:0.613:p	DHA	**<0.001**	↑EPA/ ↑DHA	↑DHA	↑	↑	82.0	82.6	73.1
Tetrahydroaldosterone glucuronide	539.249:0.472:n	DHA	0.019			↑	↑	59.1	68.0	65.4
Pyroglutamylisoleucine (pGlu-Ile)	241.120:0.653:n	DHA	<0.001 *	↑EPA/ ↑DHA				67.3	81.0	64.7
Choline	104.108:0.333:p	EPA						55.9	53.6	57.4
Glucuronic acid	193.035:0.772:n	EPA	**0.014**					62.7	60.8	62.3
Unknown dianion [M − 2H]^2−^	88.004:1.622:n	EPA						49.3	47.6	51.5
Quinic acid	191.056:0.798:n	EPA	0.035					55.1	59.5	62.6
Tiglylglycine	156.066:0.843:n	EPA	**<0.001**	↓DHA	↓DHA			69.4	71.3	60.6
Unknown cation	300.215:0.841:p	EPA	**<0.004**					52.3	68.2	66.3
*O*-Butyrylcarnitine	232.155:0.718:p		0.039					64.1	64.8	51.1
*N1*-Accetylspermidine (*N1*-AcSpm)	188.176:0.259:p		0.040			↓	↓	70.5	55.3	50.7
Unknown cation isobar#2	258.110:0.788:p	EPA	0.049					49.6	59.0	57.2
Pyroglutamylleucine (pGlu-Leu)	241.120:0.662:n		0.002 *	↑EPA/ ↑DHA				70.9	75.9	65.4
Symmetric dimethylarginine	203.151:0.478:p						↑	57.6	56.0	44.8
Tryptophan	205.098:0.899:p						↑	68.1	54.7	63.5
Carboxybutylhomocysteine	222.080:0.762:p						↓	57.4	51.6	48.4

^1^ Putative urinary metabolite as annotated by its accurate mass, relative migration time, and ionization mode (*m*/*z*:RMT:mode). ^2^ Used O3I values from all participants at both timepoints that were either <4% or >8% (*n* = 113 from a possible 176). Individuals with an O3I value between these cut-offs were not considered in this analysis. (↑) indicates an increase in urinary metabolite by supplement group (ANOVA) or a positive association with the O3I (LASSO); (↓) indicates a decrease in urinary metabolite by supplement group (ANOVA) or an inverse relationship with the O3I (LASSO). (*) indicates *p* value for Kruskal–Wallis test or significant (*p* < 0.05) Dunn’s pairwise test at 12 weeks. Significant P_int_ values after Bonferroni control for multiple testing for 2-way repeated measures ANOVA are indicated in bold font.

## Data Availability

The datasets presented in this study are available upon request to the corresponding author. The data are not publicly available due to ethical restrictions.

## Supplementary Materials

The following supporting information can be downloaded at: https://www.mdpi.com/article/10.3390/metabo13101071/s1, Table S1: Full annotation of the 17 urinary metabolites significantly associated with n3-LCPUFA supplementation or the O3I reported in Table 3; Table S2: glmmLasso selected urinary metabolites on the Omega-3 Index (O3I) and EPA + DHA (ng/mg Hb) integrated datasets; Figure S1: PCA plots of urinary metabolites and erythrocyte fatty acids; Figure S2: sPLS-DA loading weights of top 15 urinary metabolites for component 1 and 2; Figure S3: Tentative identification of CPCA based on spiking into pooled urine when using MSI-CE-MS; Figure S4: Annotation and tentative identification of unknown anion isomers pyroglutamylisoleucine (pGlu-Ile) and pyroglutamylleucine (pGlu-Leu); Figure S5: Annotation and tentative identification of an unknown dianion (221.075:0.927:n; [M − 2H]^2−^); Figure S6: Estimate values for glmmLasso selected urinary metabolites with the O3I and EPA + DHA ng/mg Hb as outcome variables; Figure S7: Annotation and tentative identification of an unknown cationic urinary metabolite *N1*-acetylspermidine (*N1*-AcSpm); Figure S8: Correlation plots between the top two urinary metabolites and the O3I and EPA + DHA ng/mg Hb.
