# Supplementary material for: Urinary Metabolite Profiling to Non-Invasively Monitor the Omega-3 Index: An Exploratory Secondary Analysis of a Randomized Clinical Trial in Young Adults
Source: Metabolites. 2023 Oct 12;13(10):1071. doi: 10.3390/metabo13101071 (PMC10608872; doi:10.3390/metabo13101071)
Supplement: Supplementary file 1 [file metabolites-13-01071-s001.zip › metabolites-2629999-supplementary.pdf]

# SUPPLEMENTARY FILE

## Urinary metabolite profiling to non-invasively monitor the omega-3 index: An exploratory secondary analysis of a randomized clinical trial in young adults.

Brittany C. MacIntyre[1], Meera Shanmuganathan[2], Shannon L. Klingel[1], Adrian Chabowski[3], Zachary Kroezen[2], Erick Helmeczi[2], Na-Yung Seoh[2], Vanessa Martinez[2], Zeny Feng[4], Philip Britz-McKibbin[2,*], and David M. Mutch[1,*]

[1]*Department of Human Health and Nutritional Sciences, University of Guelph, Guelph, Ontario, Canada*
[2]*Department of Chemistry and Chemical Biology, McMaster University, Hamilton, Ontario, Canada*
[3]*Department of Physiology, Medical University of Bialystok, Bialystok, Poland*
[4]*Department of Mathematics & Statistics, University of Guelph, Guelph, Ontario, Canada*

[*]**Corresponding Authors**:

P. Britz-McKibbin, McMaster University, Department of Chemistry and Chemical Biology, 1280 Main St. W., Hamilton, ON, Canada L8S 4L8 (e-mail: britz@mcmaster.ca)

D.M. Mutch, University of Guelph, Department of Human Health & Nutritional Science, 50 Stone Rd. E., Guelph, ON, Canada N1G 2W1 (e-mail: dmutch@uoguelph.ca)

---

**Table S1**: Full annotation of the 17 urinary metabolites significantly associated with n3-LCPUFA supplementation or the O3I reported in Table 3.

| Metabolite/Identifier[1] | *m/z*:RMT:mode[2] | Molecular Formula[2] | Mass Error[2] (ppm) |
|---|---|---|---|
| Choline | 104.108:0.333:p | $C_5H_{14}NO$ | 2.9 |
| Tiglylglycine (HMBD0000959) | 156.066:0.843:n | $C_7H_{11}NO_3$ | 4.5 |
| *S*-Carboxypropylcysteamine (HMDB0002169) | 164.074:0.613:p | $C_6H_{13}NO_2S$ | 1.8 |
| *N1*-Acetylspermidine (HMDB0001276) | 188.175:0.259:p | $C_9H_{21}N_3O$ | 6.4 |
| Quinic acid (HMDB0003072) | 191.056:0.798:n | $C_7H_{12}O_6$ | 4.2 |
| Glucuronic acid (HMDB0000127) | 193.035:0.772:n | $C_6H_{10}O_7$ | 2.6 |
| Tryptophan | 205.098:0.899:p | $C_{11}H_{12}N_2O_2$ | 2.9 |
| Unknown dianion $[M-H_2]^{2-}$ | 221.075:0.927:n | $C_{22}H_{28}N_4O_2S_2$ | -- |
| Carboxybutylhomocysteine | 222.080:0.762:p | $C_8H_{15}NO_4S$ | 2.7 |
| *O*-Butyrylcarnitine (HMDB0002013) | 232.155:0.718:p | $C_8H_{10}NO_4$ | 4.7 |
| Pyroglutamylisoleucine (HMDB0341381) | 241.120:0.653:n | $C_{11}H_{18}N_2O_4$ | 6.7 |
| Pyroglutamylleucine (HMDB0341382) | 241.120:0.662:n | $C_{11}H_{18}N_2O_4$ | 3.0 |
| Unknown cation isobar#2 | 258.110:0.788:p | $C_{11}H_{19}N_3S_2$ | -- |
| Unknown cation | 300.215:0.841:p | $C_{17}H_{25}N_5$ | -- |
| Tetrahydroaldosterone glucuronide (HMDB0010357) | 539.249:0.472:n | $C_{27}H_{40}O_{11}$ | 0.6 |
| Unknown dianion $[M-H_2]^{2-}$ | 88.004:1.622:n | $C_4H_6N_2O_6$ | 5.7 |
| Symmetric dimethylarginine | 203.151:0.478:p | $C_8H_{18}N_4O_2$ | 3.4 |

[1]*Putative urinary metabolite identified together with their corresponding HMDB ID# if reported.*
[2]*All urinary metabolites were annotated based on their accurate mass, relative migration time and ionization mode (m/z:RMT:mode), most likely molecular formulae, and mass error (if structure known).*

**Table S2**: glmmLasso selected urinary metabolites on the Omega-3 Index (O3I) and EPA+DHA (ng/mg Hb) integrated datasets.

| | | O3I Outcome | | EPA+DHA (ng/mg Hb) Outcome | |
|---|---|---|---|---|---|
| **Metabolite[1]** | ***m/z*:RMT:mode** | **Estimate** | **P value** | **Estimate** | **P value** |
| Unknown dianion $[M-H_2]^{2-}$ | 221.068:0.927:n | 1.079 | < 0.001 | 0.832 | < 0.001 |
| S-Carboxypropylcysteamine | 164.074:0.613:p | 0.501 | < 0.001 | 0.535 | < 0.001 |
| Tetrahydroaldosterone glucuronide | 539.249:0.472:n | 0.365 | < 0.001 | 0.264 | < 0.001 |
| *N1*-Acetylspermidine | 188.175:0.259:p | −0.538 | < 0.001 | −0.858 | < 0.001 |
| Symmetric dimethylarginine | 203.151:0.478:p | - | - | 0.551 | < 0.001 |
| Tryptophan | 205.098:0.899:p | - | - | 0.166 | < 0.001 |
| Carboxybutylhomocysteine | 222.080:0.762:p | - | - | −0.234 | < 0.001 |

[1]*Putative urinary metabolite as annotated by its accurate mass, relative migration time and ionization mode (m/z:RMT:mode). Most likely molecular formulae are provided for unknown chemical structures.*

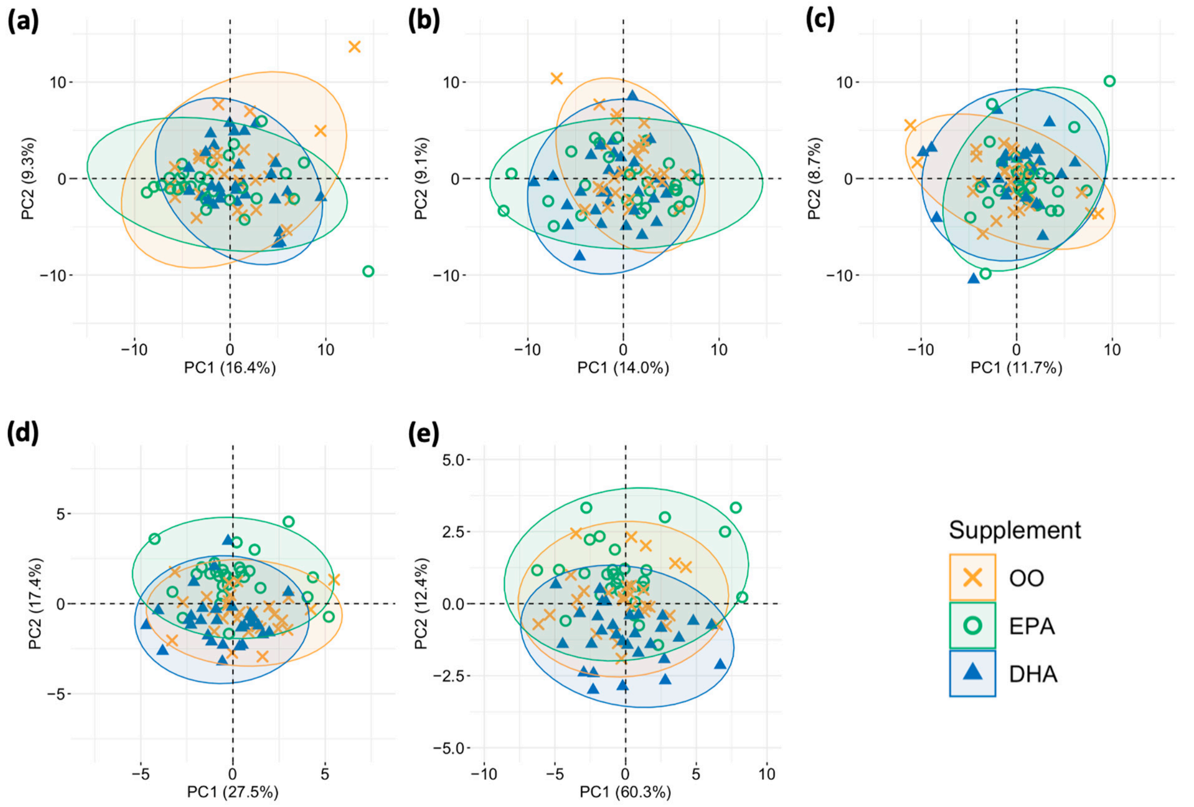


**Figure S1.** PCA plots of (**a**) baseline and (**b**) final (12 weeks) of 124 creatinine-normalized urinary metabolites for control (OO), EPA, and DHA groups (OO = orange, EPA = green, DHA = blue). Total variation explained in PC1 and PC2 plots was 25.7% and 23.1% respectively for each time point (**a, b**). (**c**) PCA plots for 88 participant delta values of 124 urinary metabolites using RPA; (**d**) 15 qualitative FA measurements (relative % FA), and (**e**) 15 quantitative FA measurements (ng FA/mg Hb). Total variation explained in PC1 and PC2 plots was 20.4%, 44.9%, 72.7% respectively for each dataset (**c-e**).

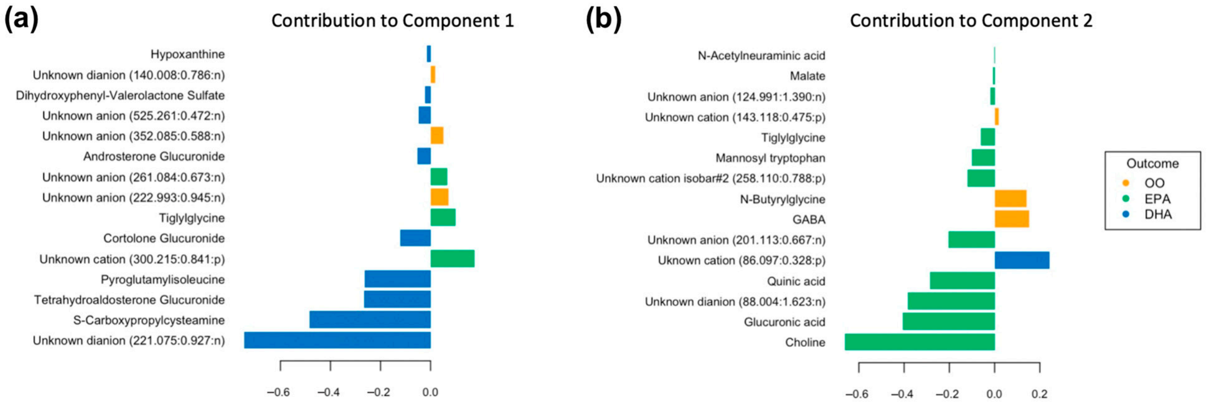


**Figure S2**. sPLS-DA loading weights reflecting the contribution for 15 urinary metabolites on (**a**) PC1 and (**b**) PC2 coloured by group (OO = orange, EPA = green, DHA = blue). All unknown metabolites are annotated based on their characteristic *m/z*:RMT:mode.

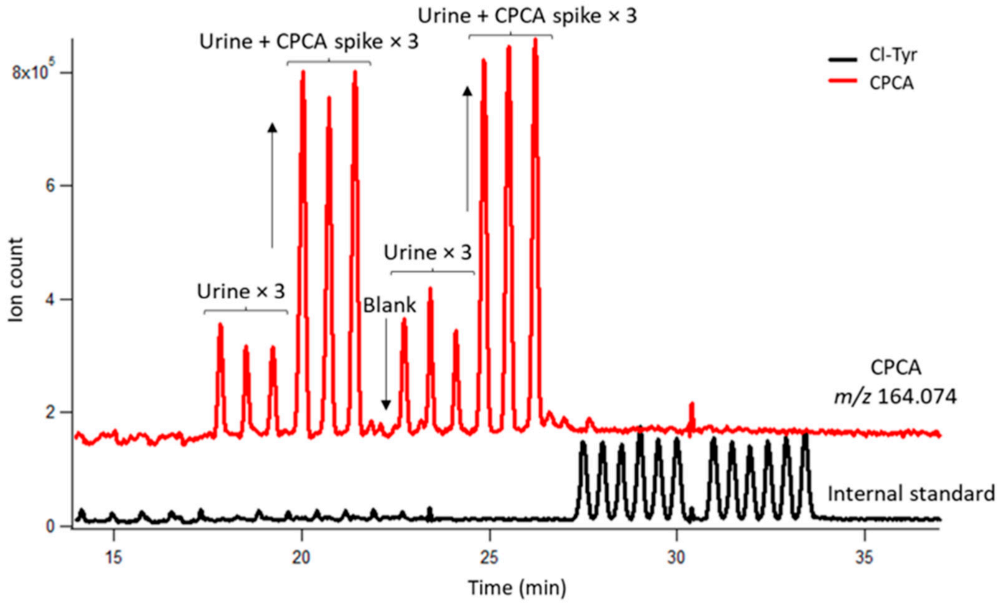


**Figure S3.** Tentative identification of CPCA based on spiking into pooled urine when using MSI-CE-MS with a 13 serial sample injection format, which confirmed its co-migration with the unknown sulfur-containing metabolite (*m/z* 164.074) under positive ion mode conditions.

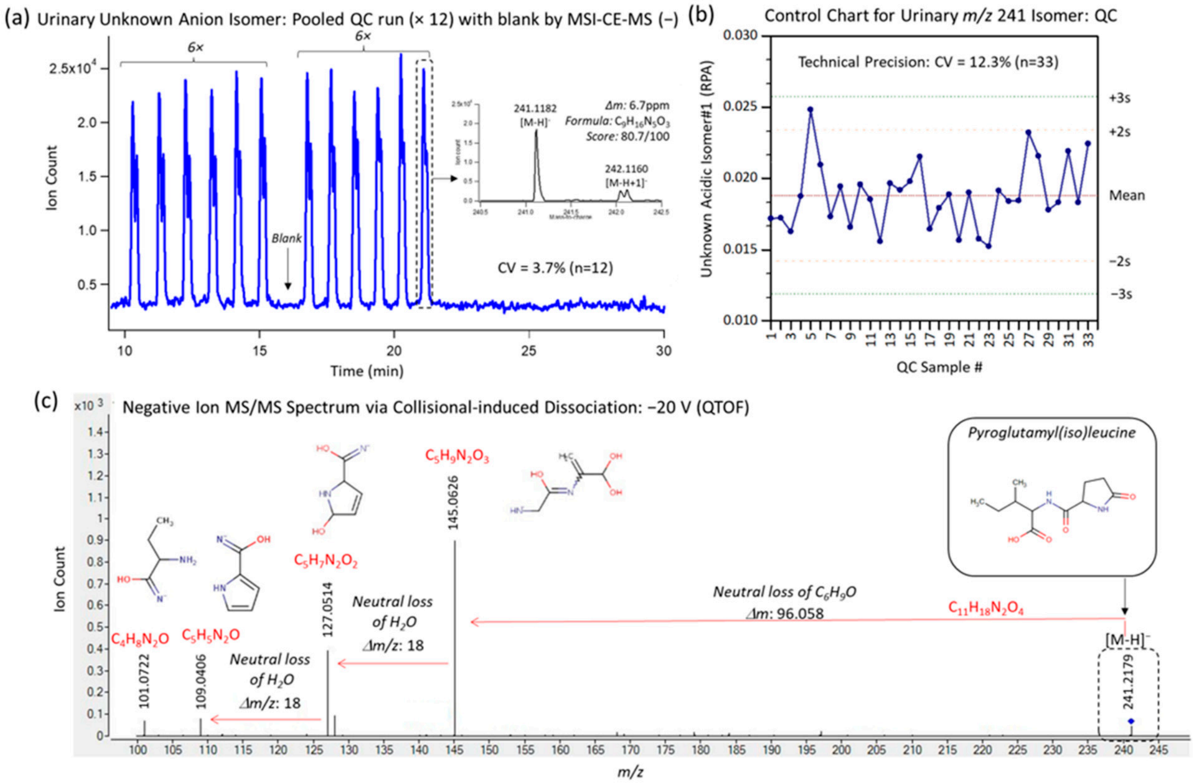


**Figure S4.** Tentative identification of two unknown anionic urinary metabolites that migrate as partially resolved isomers by MSI-CE-MS under negative ion mode conditions. (**a**) Representative extracted ion electropherogram (EIE) when using MSI-CE-MS under negative ion mode for the unknown anion isomer highlighting a serial injection of 12 urine samples and a blank in a single run. The first migrating isomer (241.120:0.653:n) was the determined as the third most significant urinary metabolite associated with the O3I notably following DHA ingestion. (**b**) Technical precision of first migrating unknown anion isomer in QC samples (CV = 12.3%, n = 33). (**c**) Annotation of the first migrating unknown anion isomer MS/MS at an optimal collision energy, tentatively identified as pyroglutamylisoleucine (pGlu-Ile), whereas its slower migrating isomer is likely pyroglutamylleucine (pGlu-Leu).

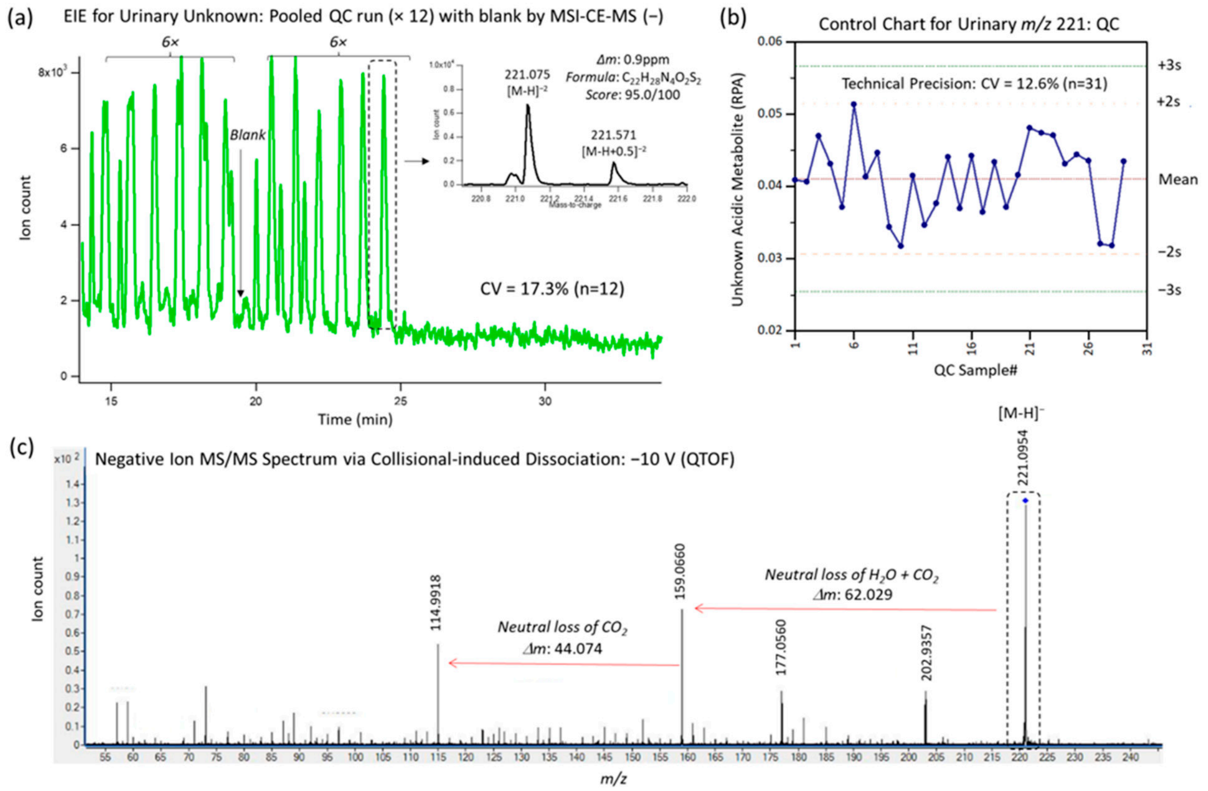


**Figure S5.** Annotation of an unknown urinary dianion (221.075:0.927:n; $[M{-}2H]^{2-}$) based on its (**a**) extracted ion electropherogram (EIE) when using MSI-CE-MS under negative ion mode demonstrating a charge state (i.e., isotopic pattern) that was associated with DHA supplementation. Although this acidic metabolite was measured with (**b**) adequate technical precision in QC samples (CV < 15%, n = 31), its low abundance prevented acquisition of a good quality MS/MS spectra under negative ion mode conditions. (**c**) Collisional-induced dissociation experiments confirmed the likely presence of two acidic functional moieties (-COOH) based on corresponding neutral mass losses.

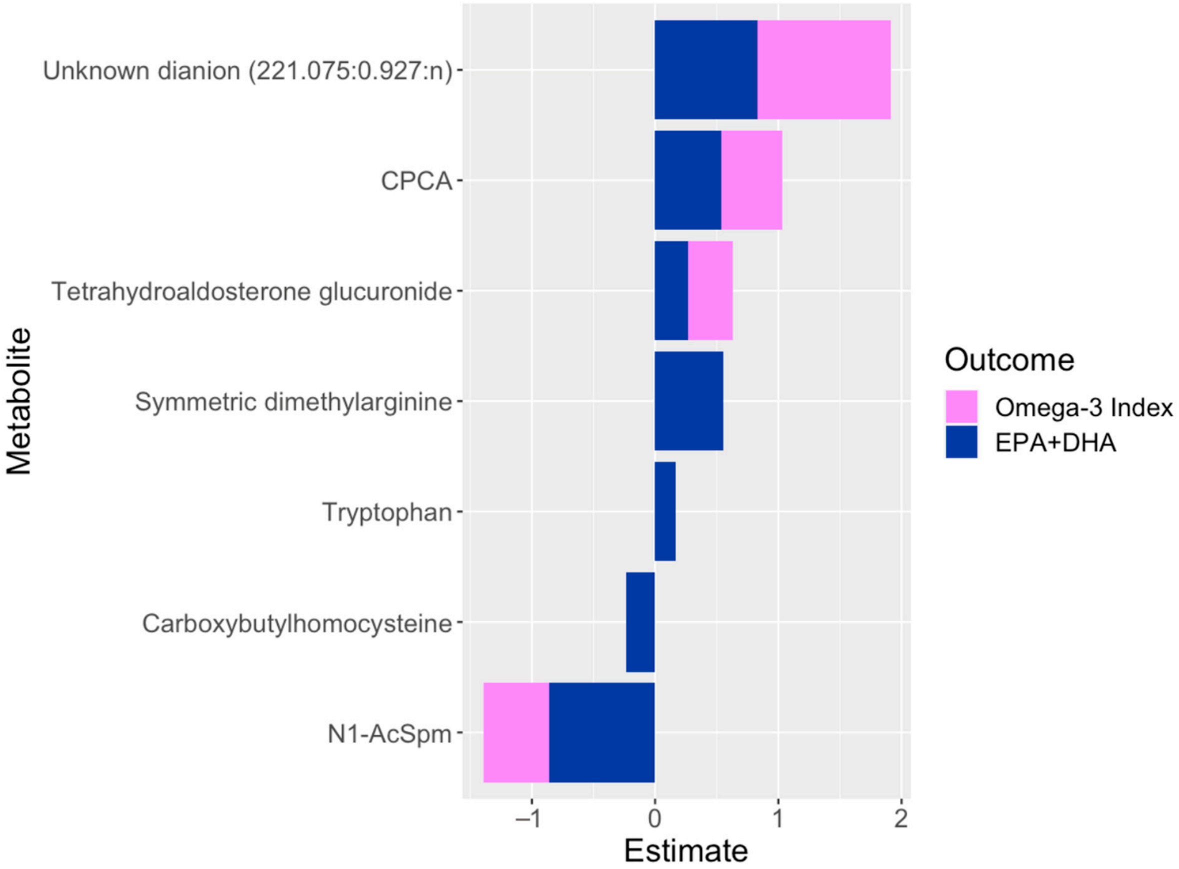


**Figure S6.** Estimate values for glmmLasso urinary metabolites from two integrated datasets with scaled from 0-10 or 80-700 urine metabolites as covariates and the O3I or EPA+DHA (ng/mg Hb), respectively, as outcome variables. Urinary metabolites with a positive estimate result indicate a positive association with the outcome variable and metabolites with a negative estimate result indicate a negative association with the outcome variable. Estimates from values from the O3I (pink) and EPA+DHA (ng/mg Hb) (navy) are depicted in stacked bars. Unknown metabolites were annotated based on their characteristic m/z:RMT:mode. CPCA = 2-Carboxypropylcysteamine, *N1*-AcSpm = *N1*-Acetylspermidine.

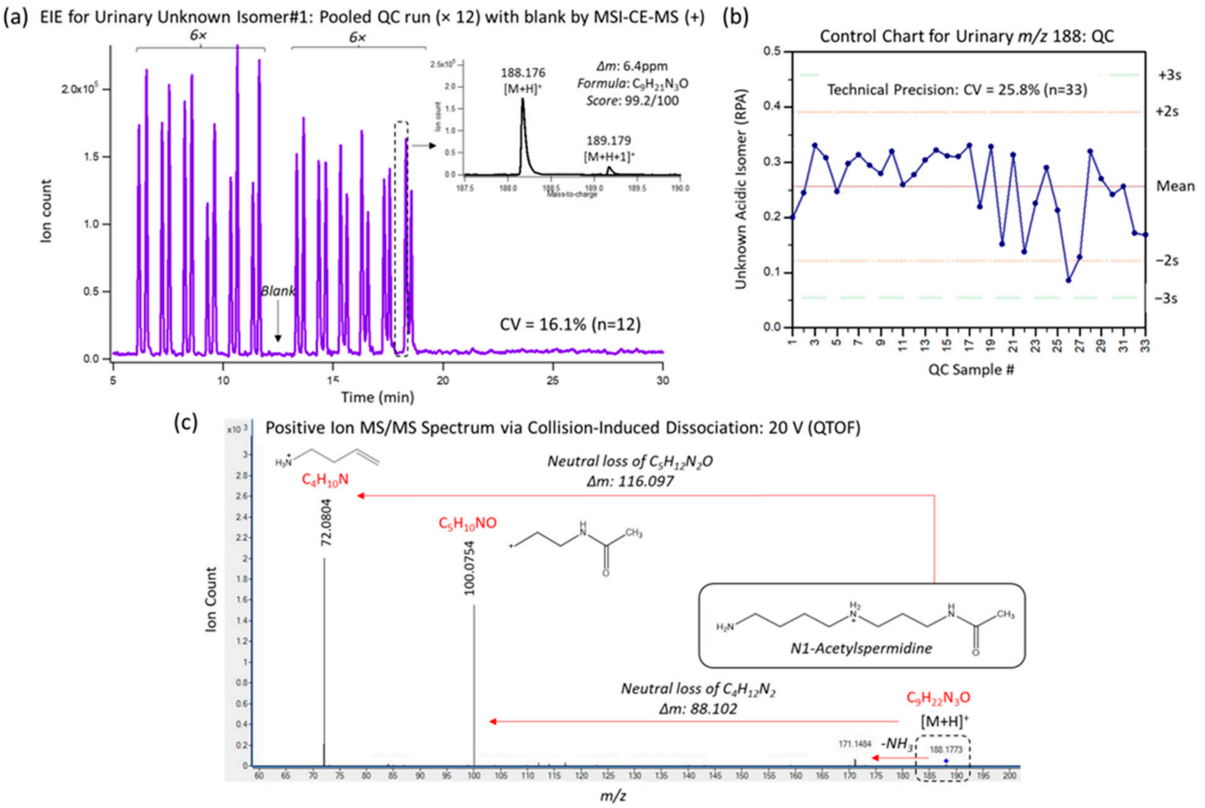


**Figure S7.** Tentative identification of two unknown cationic urinary metabolites that (**a**) migrate as partially resolved isomers by MSI-CE-MS under positive ion mode conditions. However, only the first migrating isomer (188.175:0.259:p) was associated with the O3I. (**b**) This unknown isomer was measurable with acceptable technical precision in QC samples. (**c**) Annotation of this first migrating isomer MS/MS at an optimal collision energy, tentatively identified as *N1*-acetylspermidine (*N1*-AcSpm), whereas its slower migrating isomer is likely *N8*-acetylspermidine (*N8*-AcSpm).

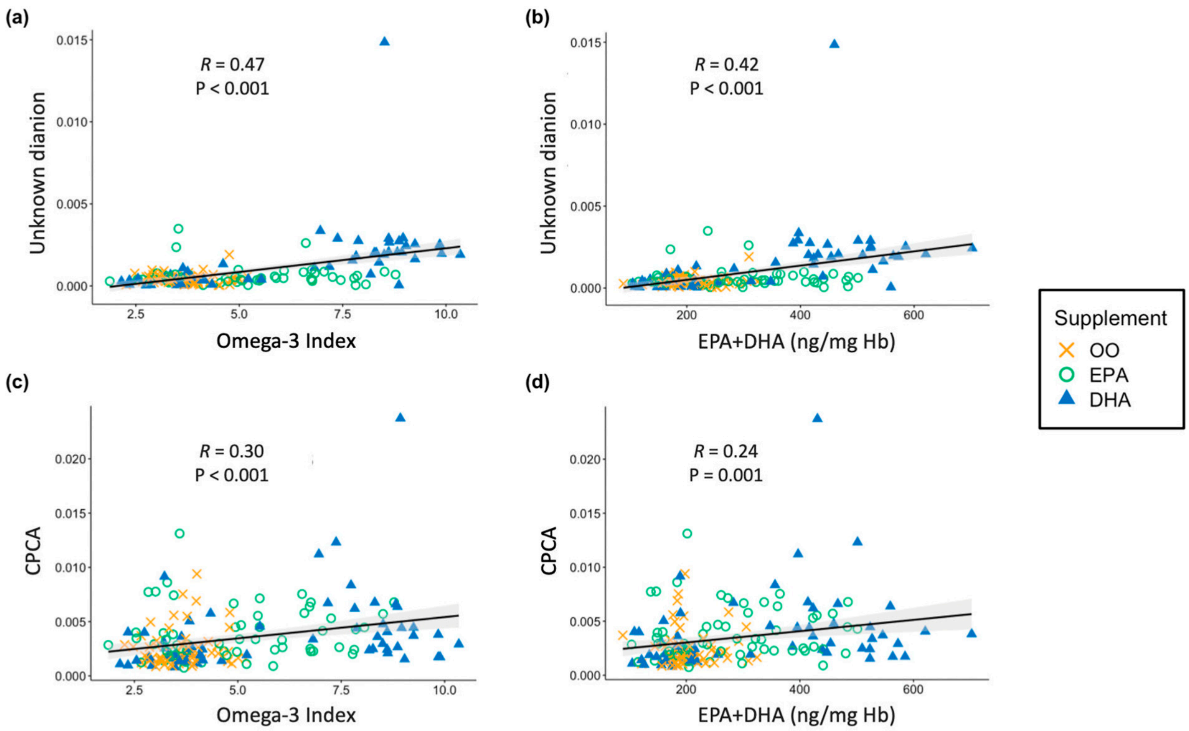


**Figure S8.** Correlation plots for the (**a**) unknown dianion (221.075:0.927:n) and the O3I (R = 0.47, P < 0.001); (**b**) unknown dianion (221.075:0.927:n) and the sum of EPA+DHA (ng/mg Hb) (R = 0.042, P < 0.001); (**c**) *S*-carboxypropylcysteamine (CPCA; 164.074:0.613:p) and the O3I (R = 0.30, P < 0.001); and (**d**) CPCA and the sum of EPA+DHA (ng/mg Hb) (R = 0.24, P = 0.001). OO = yellow, EPA = green, DHA = blue.
